# Highly Potent and Selective Dopamine D_4_ Receptor Antagonists Potentially Useful for the Treatment of Glioblastoma

**DOI:** 10.1021/acs.jmedchem.2c00840

**Published:** 2022-09-13

**Authors:** Pegi Pavletić, Ana Semeano, Hideaki Yano, Alessandro Bonifazi, Gianfabio Giorgioni, Alessandro Piergentili, Wilma Quaglia, Maria Giovanna Sabbieti, Dimitrios Agas, Giorgio Santoni, Roberto Pallini, Lucia Ricci-Vitiani, Emanuela Sabato, Giulio Vistoli, Fabio Del Bello

**Affiliations:** † Scuola di Scienze del Farmaco e dei Prodotti della Salute, Università di Camerino*,*, Camerino 62032, Italy; ‡ Department of Pharmaceutical Sciences, School of Pharmacy and Pharmaceutical Sciences, Bouvé College of Health Sciences, Center for Drug Discovery, Northeastern University, Boston, Massachusetts 02115, United States; § Medicinal Chemistry Section, Molecular Targets and Medications Discovery Branch, National Institute on Drug Abuse−Intramural Research Program, National Institutes of Health, 333 Cassell Drive, Baltimore, Maryland 21224, United States; ∥ Dipartimento di Scienze Farmaceutiche, Università degli Studi di Milano, Via Mangiagalli 25, Milano 20133, Italy; ⊥ Scuola di Bioscienze e Medicina Veterinaria, Università di Camerino, Via Gentile III da Varano, Camerino 62032, Italy; # Institute of Neurosurgery, Scientific Hospitalization and Care Institute (IRCCS), Gemelli University Polyclinic Foundation, Rome 00168, Italy; ¶ Institute of Neurosurgery, School of Medicine, Catholic University, Rome 00168, Italy; ∇ Department of Hematology, Oncology and Molecular Medicine, Istituto Superiore di Sanità, Rome 00161, Italy

## Abstract

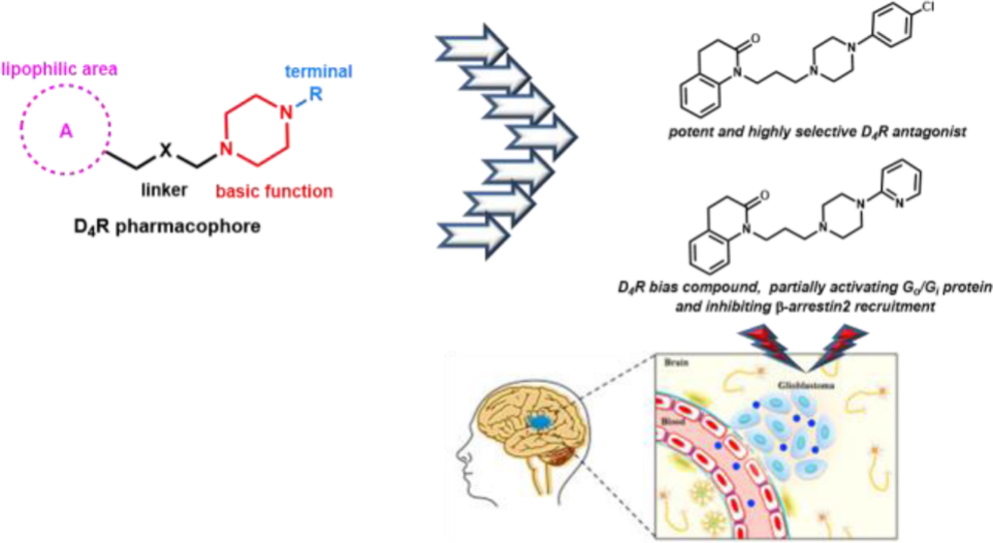

To better understand the role of dopamine D_4_ receptor (D_4_R) in glioblastoma (GBM), in the present paper, new ligands endowed with high affinity and selectivity for D_4_R were discovered starting from the brain penetrant and D_4_R selective lead compound 1-(3-(4-phenylpiperazin-1-yl)propyl)-3,4-dihydroquinolin-2(1*H*)-one (**6**). In particular, the D_4_R antagonist **24**, showing the highest affinity and selectivity over D_2_R and D_3_R within the series (D_2_/D_4_ = 8318, D_3_/D_4_ = 3715), and the biased ligand **29**, partially activating D_4_R G_i_-/G_o_-protein and blocking β-arrestin recruitment, emerged as the most interesting compounds. These compounds, evaluated for their GBM antitumor activity, induced a decreased viability of GBM cell lines and primary GBM stem cells (GSC#83), with the maximal efficacy being reached at a concentration of 10 μM. Interestingly, the treatment with both compounds **24** and **29** induced an increased effect in reducing the cell viability with respect to temozolomide, which is the first-choice chemotherapeutic drug in GBM.

## Introduction

Dopamine (DA) is a catecholamine neurotransmitter that mediates a wide variety of functions via binding with five dopamine receptor subtypes (DRs), belonging to class-A G protein-coupled receptor (GPCR) family. The binding site of DA is located in the extracellular region of DRs between the transmembrane (TM) helices. Based on structural characteristics, DRs are divided into two subfamilies, namely, D_1_-like receptors, comprising D_1_R and D_5_R, and D_2_-like receptors, including D_2_R, D_3_R, and D_4_R.^1−4^ After DA binding, D_1_-like receptors activate stimulatory G-proteins (Gα_s/olf_) and upregulate intracellular levels of adenosine 3′,5′-cyclic monophosphate (cAMP) by stimulating adenylyl cyclase (AC). Differently, D_2_-like receptors activate inhibitory G-proteins (Gα_i/o_) and downregulate the AC activity.^5,6^ Moreover, DRs have demonstrated to modulate other G-protein-dependent or -independent pathways, involving protein kinases, ion channels, phospholipases, and β-arrestins.^4,7^

Within the D_2_-like subfamily, D_4_R has recently emerged as an attractive target for the management of widespread diseases, including cancer, alcohol/substance use disorders, attention deficit hyperactive disorder, and eating disorders.^8−10^ This subtype is characterized by high polymorphism in the human genome^2^ and in particular, in the gene region codifying for the third intracellular loop (ICL_3_) of the receptor. Indeed, the ICL_3_ of D_4_R contains from 2- to 11-repeat forms of a 16-amino acid polypeptide, with the most common versions being 4-repeat (64%) followed by 7- and 2-repeat (21 and 8%, respectively). This polymorphism can influence the coupling of D_4_R to AC.^4,9,11,12^

**Figure 1:**
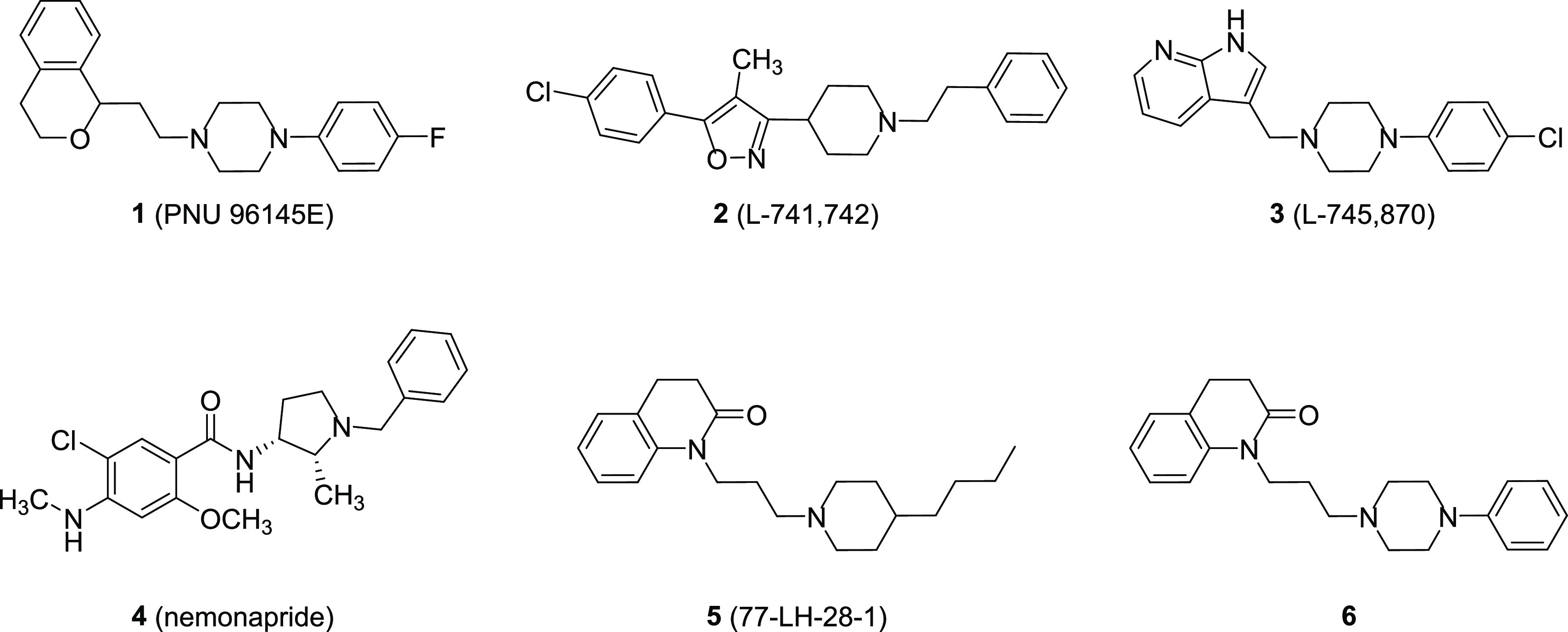
Chemical structures of compounds **1**–**6**.

D_4_R subtype is predominantly expressed in the central nervous system (CNS), especially in the frontal cortex, medulla, hippocampus, hypothalamus, pituitary gland, and amygdala.^13,14^ D_4_R expression is weak when compared to that of the other dopamine receptors,^15^ but its anatomical localization in the prefrontal cortex strongly indicates the role of this subtype in cognition and emotions. Moreover, neurobiological evidence suggest a possible relationship between D_4_R and glioblastoma (GBM)^16,17^ and particularly, D_4_R antagonists have proved to selectively inhibit GBM growth with a lower effect on the cell viability of normal neural stem cells. The D_4_R antagonists PNU 96415E (**1**) and L-741,742 (**2**) (Figure 1) have been demonstrated to disrupt the autophagy-lysosomal pathway specifically in GBM neural stem cells, inhibiting their survival and proliferation.^17^

The resolved crystal structures of the complexes between D_4_R and the potent antagonist L-745,870 (**3**) (PDB ID = 6IQL)^18^ or the antipsychotic drug nemonapride (**4**) (PDB ID = 5WIU)^19^ (Figure 1) have greatly ameliorated the knowledge of the molecular mechanisms related to the D_4_R modulation.

We have recently demonstrated that the known M_1_ muscarinic bitopic agonist 77-LH-28-1 (**5**, Figure 1)^20^ also behaved as a potent D_4_R antagonist and showed an unexpected D_4_R selectivity with respect to D_2_R and D_3_R (p*K*_i_ D_2_R = 6.17; D_3_R = 6.21; and D_4_R = 9.01).^21^ Compound **5** was taken as a starting point for a structure–activity relationship (SAR) study, which led to the discovery of its analogue **6** (Figure 1) characterized by a 4-phenylpiperazine group instead of the 4-butylpiperidine moiety of **5**. Compound **6** maintained high affinity for D_4_R (p*K*_i_ = 8.54) and showed high selectivity not only over D_2_R and D_3_R (selectivity ratio D_2_/D_4_ = 380 and D_3_/D_4_ = 457) but also over other receptors and transporters. In functional assays, it showed a biased profile behaving as a partial agonist for D_4_R-G_i_ protein activation and as an antagonist for β-arrestin recruitment. Moreover, it demonstrated to be highly brain penetrant in mice.

**Figure 2:**
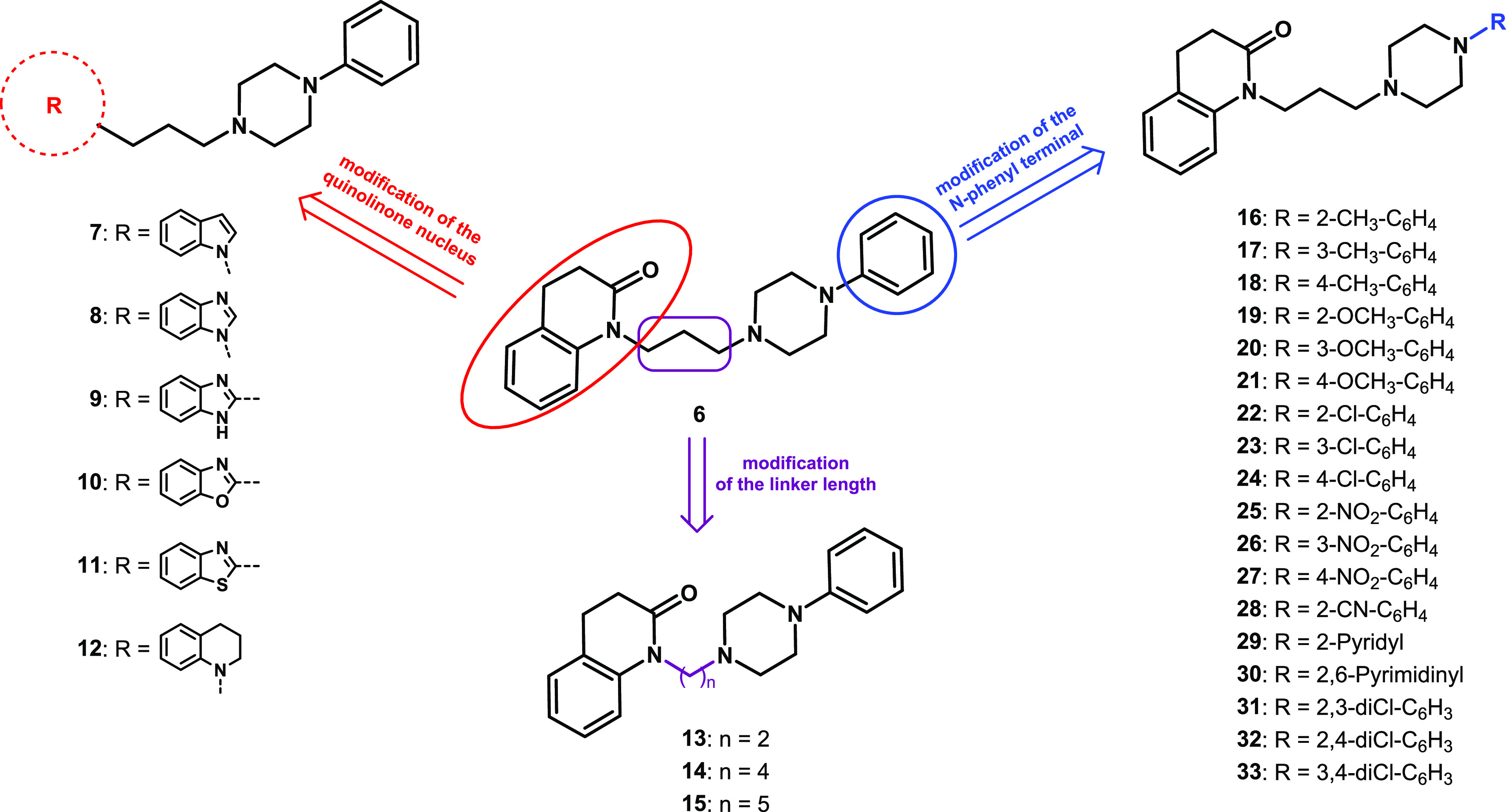
Modifications of the chemical structure of the lead compound **6** yielding derivatives **7–33**.

Therefore, due to its promising profile, in the present study, **6** has been chosen as a lead compound for the discovery of new potent and selective D_4_R ligands useful as pharmacological tools to better understand the role of D_4_R in GBM. In particular, maintaining the *N*-arylpiperazine moiety, a well-known scaffold of potent D_4_R ligands,^8,10^ including **1** and **3**, the following modifications were designed: (i) replacement of the quinolinone portion with other bioisosteric nuclei (compounds **7–12**, Figure 2), whose choice was inspired by D_4_-selective ligands known in the literature;^8^ (ii) replacement of the propyl linker with chains of different lengths (compounds **13–15**, Figure 2), to evaluate the role of the distance between the basic function and the tetrahydroquinolinone nucleus; (iii) introduction of substituents with different electronic and lipophilic contributions in all combinations, such as CH_3_(+π, −σ), OCH_3_(−π, −σ), Cl(+π, +σ), and NO_2_(−π, +σ), in ortho-, meta-, and para-positions of the *N*-phenyl ring (compounds **16–27**, Figure 2).^22^

Compounds **28–30** (Figure 2), bearing 2-cyanophenyl, 2-pyridyl, and 2-pyrimidinyl terminals, that are present in known potent and selective D_4_R ligands,^8^ as well as the 2,3-, 2,4-, and 3,4-dichlorophenyl derivatives **31**–**33** (Figure 2) were also prepared. All the compounds were evaluated for their affinity at D_2_R, D_3_R, and D_4_R by radioligand binding assays. Although compounds **7**,^23^**8**,^24^**14**,^25^**19**,^26^**21**,^26^**23**,^27^**29**,^26^**30**,^26^ and **31**(26) had previously been reported in the literature, they had never been studied at D_4_R. The most selective D_4_R ligands were also tested for their functional activities by bioluminescence resonance energy transfer (BRET) assays to detect D_4_R G-protein activation and β-arrestin recruitment. The resolved crystal structure of the human D_4_R complexed with nemonapride (PDB Id: 5WIU)^19^ allowed to clarify the binding mode of the proposed derivatives and to support the SAR studies. Finally, the most interesting compounds were evaluated for their potential in affecting the viability of GBM cell lines and primary GBM stem cells (GSC#83).

## Results and Discussion

### Chemistry

**Scheme 1:**
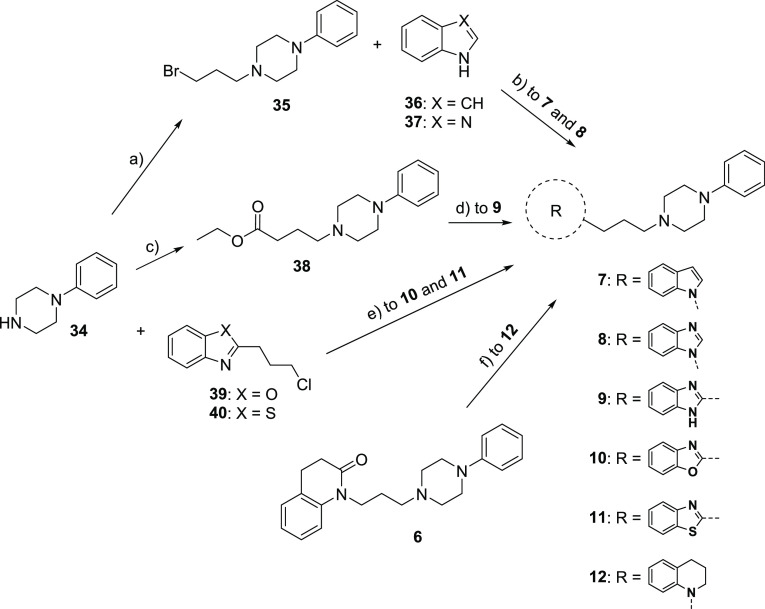
Synthesis of **7**−**12** Reagents: (a) 1,3-dibromopropane, KOH, DMSO; (b) NaH, DMF; (c) ethyl 4-bromobutanoate, NaHCO_3_, EtOH; (d) benzene-1,2-diamine, 4M HCl in dioxane; (e) K_2_CO_3_, KI, DME; (f) BH_3_·S(CH_3_)_2_, THF.

Compounds **7–12** were prepared following the procedure reported in Scheme 1. The *N*-alkylation of the commercially available 1-phenylpiperazine **34** with 1,3-dibromopropane in the presence of potassium hydroxide afforded intermediate **35**,^28^ which was treated with indole (**36**) or benzimidazole (**37**) in the presence of sodium hydride to give **7** and **8**, respectively. The reaction of **34** with ethyl 4-bromobutanoate in the presence of sodium bicarbonate yielded intermediate **38**,^29^ whose treatment with benzene-1,2-diamine led to derivative **9**. The reaction between **34** and alkyl chlorides **39**(30) or **40**(31) in the presence of potassium carbonate and potassium iodide gave compounds **10** and **11**, respectively. Amine **12** was prepared by reduction of the lead compound **6** with borane dimethyl sulfide complex.

**Scheme 2:**
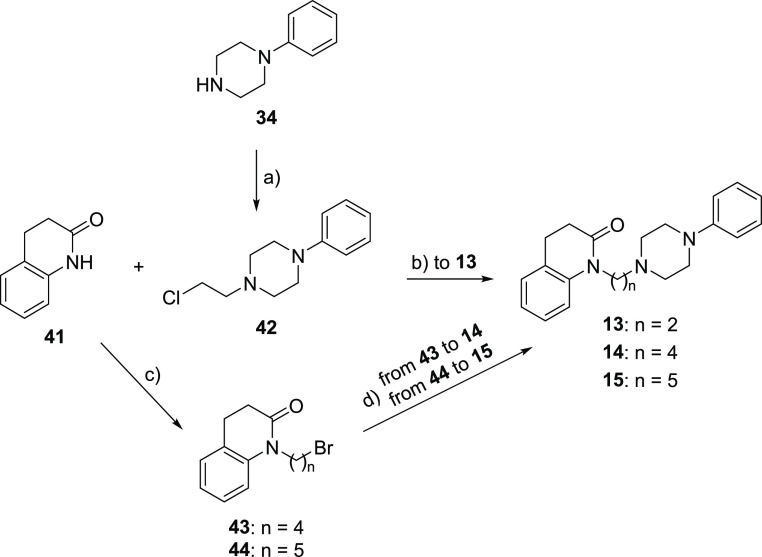
Synthesis of **13**−**15** Reagents: (a) 1-bromo-2-chloroethane, K_2_CO_3_, acetone; (b) NaH, xylene; (c) 1,4-dibromobutane for **43** or 1,5-dibromopentane for **44**, NaH, DMF; (d) **34**, K_2_CO_3_, DMF.

Compounds **13–15** were prepared following the procedure reported in Scheme 2. The *N*-alkylation of **34** with 1-bromo-2-chloroethane in the presence of potassium carbonate afforded intermediate **42**,^32^ which was reacted with the commercially available 3,4-dihydro-2(1*H*)-quinolinone **41** to give compound **13**. The reaction of **41** with 1,4-dibromobutane or 1,5-dibromopentane in the presence of sodium hydride yielded intermediates **43**(33) and **44**,^34^ whose treatment with **34** in the presence of potassium carbonate led to derivatives **14** and **15**, respectively.

**Scheme 3:**
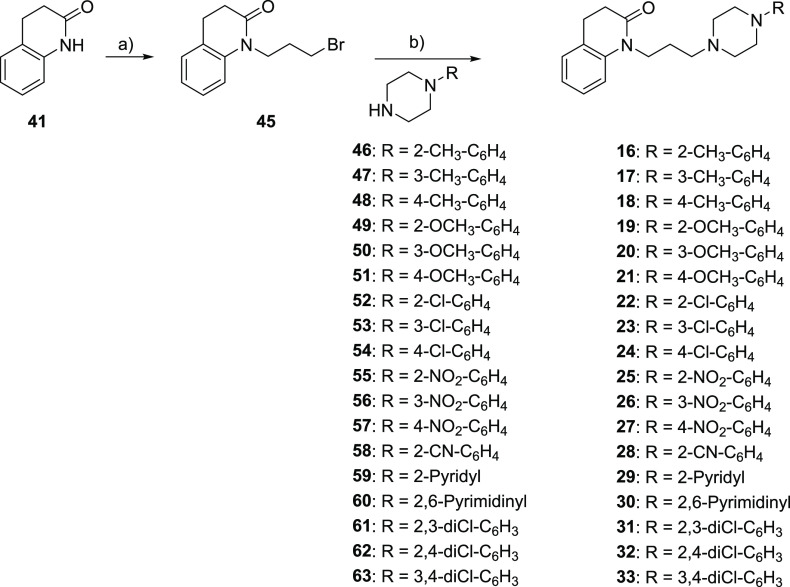
Synthesis of **16**−**33** Reagents: (a) 1,3-dibromopropane, NaH, DMF; (b) K_2_CO_3_, DMF.

The reaction of **41** with 1,3-dibromopropane in the presence of sodium hydride yielded intermediate **45**,^35^ which was treated with suitable amines **46–63** in the presence of potassium carbonate to give derivatives **16–33**, respectively (Scheme 3).

### Binding Studies

The pharmacological profile of compounds **7–33** as oxalate salts was evaluated by radioligand binding assays with human recombinant D_2_-like receptor subtypes stably expressed in HEK293T cells using the [^3^H]*N*-methylspiperone, a high-affinity D_2_-like antagonist, as radioligand to label DRs, following previously described protocols.^36,37^

D_2_R, D_3_R, and D_4_R affinity values, expressed as p*K*_i_, for ligands obtained by modifying the quinolinone nucleus (compounds **7**–**12**), the linker (compounds **13**–**15**), and the aromatic terminal (compounds **16**–**33**) of the lead compound **6** are reported in Table 1 together with those of compounds **3**, **5**, and **6**, included for useful comparison.

**Table 1:**
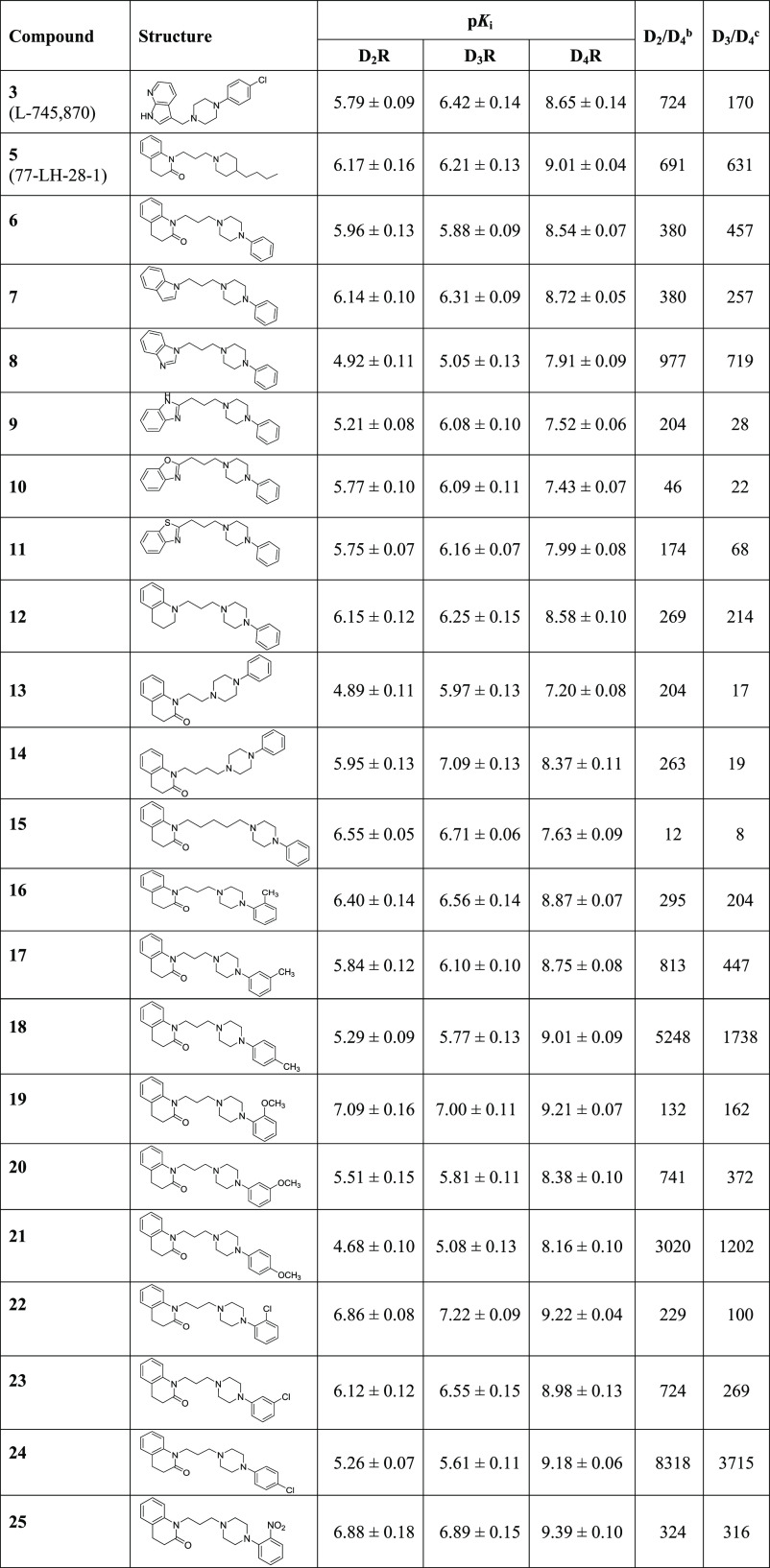

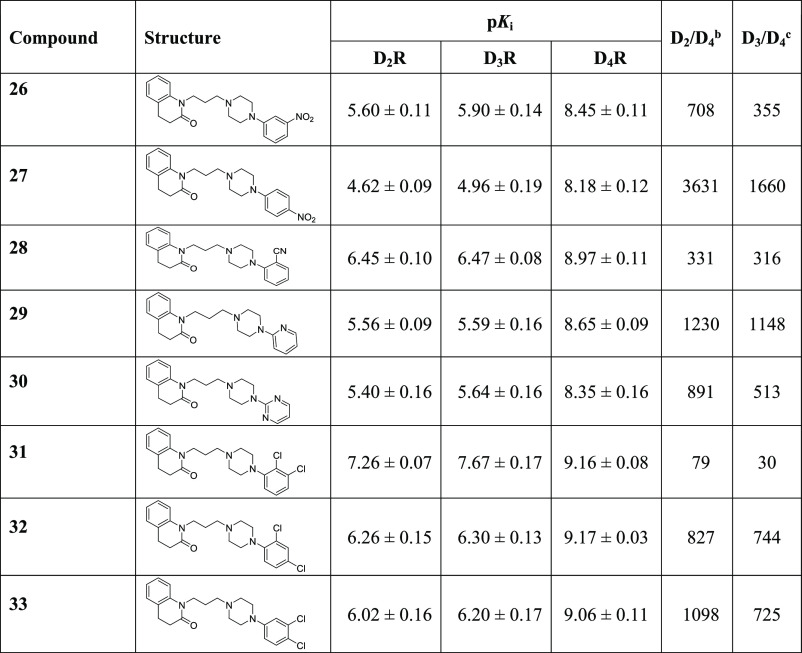
Affinity Constants, Expressed as p*K*_i_,^a^ of Compounds **3**, **5**–**33** for Human Cloned D_2L_R, D_3_R, and D_4.4_R Expressed in HEK293T Cells

a p*K*_i_ calculated from *K*_i_ values determined by competitive inhibition of [^3^H]*N*-methylspiperone binding in membranes harvested from HEK293 cells stably expressing hD_2L_R, hD_3_R, or hD_4.4_R. All values are presented as arithmetic mean ± SEM.

b Calculated as a ratio between *K*_i_ values at D_2_R and D_4_R.

c Calculated as a ratio between *K*_i_ values at D_3_R and D_4_R.

The analysis of the results highlights that, concerning the bioisosteric replacement of the tetrahydroquinolinone nucleus of **6**, all the compounds show a slightly decreased D_4_R affinity, except for the *N*-indole **7** and *N*-tetrahydroquinoline **12**, which maintain the high D_4_R affinity and selectivity of the lead. Moreover, although the *N*-benzimidazole derivative **8** binds D_4_R with lower affinity with respect to the lead compound **6**, it shows higher D_2_/D_4_ and D_3_/D_4_ selectivity ratios (D_2_/D_4_ = 380 and D_3_/D_4_ = 457 for **6**; D_2_/D_4_ = 977 and D_3_/D_4_ = 719 for **8**).

The reduction of the linker length of compound **6**, obtaining **13**, causes a marked decrease in the binding affinity only at D_4_R (p*K*_i_ D_4_R = 8.54 for **6** and p*K*_i_ D_4_R = 7.20 for **13**) and D_2_R (p*K*_i_ D_2_R = 5.96 for **6** and p*K*_i_ D_4_R = 4.89 for **13**), with a consequent decrease in a D_3_/D_4_ selectivity ratio (D_3_/D_4_ = 457 for **6** and D_3_/D_4_ = 17 for **13**). Differently, compound **14**, the higher homologue of **6** obtained by inserting a methylene unit in the linker, maintains similar D_4_R (p*K*_i_ D_4_R = 8.54 for **6** and p*K*_i_ D_4_R = 8.37 for **14**) and D_2_R (p*K*_i_ D_2_R = 5.96 for **6** and p*K*_i_ D_2_R = 5.95 for **14**) affinity values but shows an increase in D_3_R affinity (p*K*_i_ D_3_R = 5.88 for **6** and p*K*_i_ D_3_R = 7.09 for **14**). Therefore, also in this case, the D_3_/D_4_ selectivity ratio is reduced. Although compounds **13** and **14** show different affinities for D_2_R, D_3_R, and D_4_R subtypes, the D_2_/D_4_ and D_3_/D_4_ selectivity ratios are similar (D_2_/D_4_ = 204 and D_3_/D_4_ = 17, for **13** and D_2_/D_4_ = 263 and D_3_/D_4_ = 19 for **14**). Further elongation of the linker, yielding **15**, induces lower D_4_R affinity, with a consequent decrease of D_2_/D_4_ and D_3_/D_4_ selectivity ratios (12 and 8, respectively). Taken together, these results highlight that the propyl chain represents the optimal distance between the quinolinone nucleus and the basic function.

The presence of a substituent on the terminal phenyl ring of **6** markedly affects the D_2_-like affinity and selectivity profiles of the ligands. All the ortho-, meta-, and para-substituted derivatives show high D_4_R affinity. However, the derivatives **16–18** and **22–24**, bearing substituents with +π values (CH_3_ and Cl), display similar p*K*_i_ values at D_4_R regardless of their position on the phenyl ring, while substituents with -π values (OCH_3_ and NO_2_) confer to the ligands the highest affinity when they are in ortho positions (**19** vs **20** and **21** and **25** vs **26** and **27**). Interestingly, whatever the nature of the substituent is, the most D_4_R selective compounds are the para-substituted ones (**18**, **21**, **24**, and **27**) (D_2_/D_4_ = 5248 and D_3_/D_4_ = 1738 for **18**; D_2_/D_4_ = 3020 and D_3_/D_4_ = 1202 for **21**; D_2_/D_4_ = 8318 and D_3_/D_4_ = 3715 for **24**; and D_2_/D_4_ = 3631 and D_3_/D_4_ = 1660 for **27**). The improved selectivity is due to the decrease in D_2_R and D_3_R affinity when the substituent is shifted from the ortho to meta- and, especially, to para-positions.

Considering that, among the para-substituted compounds, the best selectivity profile is shown by 4-chloro derivative **24**, the influence of the dichlorophenyl disubstitution was probed by the synthesis and study of derivatives **31**–**33**. The results confirm that the presence of a substituent in the para-position of the phenyl ring is detrimental for D_2_R and D_3_R binding affinity. Indeed, the ortho/para- and meta/para-disubstituted compounds **32** and **33** show D_2_/D_4_ and D_3_/D_4_ selectivity ratios significantly higher than those of the ortho/meta-disubstituted compound **31**.

To extend the SARs concerning the aromatic terminal, the phenyl ring was replaced by other aromatic pendants, such as 2-cianophenyl (**28**), 2-pyridyl (**29**), and 2,6-pyrimidinyl (**30**) rings, which are also present in known potent and selective D_4_R ligands. Compounds **28**–**30** show D_4_R affinity values similar to that of the lead compound **6**. Moreover, **29** and **30** exhibit a slight reduction in affinity for D_2_R and D_3_R subtypes and, consequently, are more selective for D_4_R with respect to **6**. In particular, the 2-pyridyl derivative **29** shows the best selectivity profile (D_2_/D_4_ = 1230, D_3_/D_4_ = 1148).

It has been observed that ortho-, meta-, and para-regiosubstitutions on the terminal aryl ring might modulate efficacy at D_4_R of arylpiperazines.^38,39^ However, previously reported D_4_R partial or highly efficacious agonists demonstrated to bind more readily when in competition against an agonist radioligand (i.e., [^3^H]-7-OH-DPAT) instead of the classic antagonist [^3^H]*N*-methylspiperone. On the other hand, antagonists showed <10-fold difference in binding *K*_i_, or almost no difference at all, independently from the radioligand used.^39^ Based on these observations, the D_4_R affinity of the ortho-, meta-, and para-chlorophenylpiperazines **22**–**24** has also been assessed using the agonist radioligand [^3^H]-7-OH-DPAT. All the compounds did not show any major shift in their p*K*_i_ values when tested in the agonist-radioligand mode(**22**: p*K*_i_ = 9.29 ± 0.06; **23**: p*K*_i_ = 9.11 ± 0.12; and **24**: p*K*_i_ = 8.83 ± 0.11) compared to the already reported affinity obtained with [^3^H]*N*-methylspiperone (**22**: p*K*_i_ = 9.22 ± 0.04; **23**: p*K*_i_ = 8.98 ± 0.13; and **24**: p*K*_i_ = 9.18 ± 0.06), suggesting that they might behave as D_4_R antagonists.

### Functional Assays

Based on their remarkable D_4_R affinity/selectivity profiles, compounds **18**, **21**, **24**, **27**, and **29** were selected to be evaluated for their functional activities in BRET-based assays at D_4_R. Unfortunately, **27** seemed to have an intrinsic light absorption property that interfered with BRET and, therefore, it was not possible to determine its functional profile. The potencies and efficacies, expressed as pEC_50_ (−log EC_50_) and E_max_ (maximum efficacy), respectively, of **18**, **21**, **24**, and **29** are reported in Table 2 along with those of DA (D_4_R full agonist) and **3** (L745,870, D_4_R antagonist) as reference compounds.

**Table 2:** Potency (Expressed as pEC_50_^a^ or pIC_50_^a^) and Efficacy Values (%^a^, Normalized to Dopamine *E*_max_) of Dopamine (DA) and Compounds **3** (L745,870), **18**, **21**, **24**, and **29** for D_4_R Expressed in HEK293T Cells

	*G*_o_ activation (*n* ≥ 5)		*G*_i_ activation (*n* ≥ 5)		β-arrestin2 recruitment (*n* ≥ 5)	
	pEC_50_ (pIC_50_)	*E*_max_(Im_ax_)	pEC_50_(pIC_50_)	*E*_max_(*I*m_ax_)	pEC_50_(pIC_50_)	*E*_max_(I_max_)
DA	7.83 ± 0.09	100 ± 2.8	7.68 ± 0.15	100 ± 5.1	6.57 ± 0.24	100 ± 5.7
**3**	(6.84 ± 0.18)	(−75.4 ± 4.7)	(5.71 ± 0.3)	(−83.9 ± 16.3)	(6.79 ± 0.30)	(−89.4 ± 9.3)
**18**	ND (6.48 ± 0.20)	0 (−91.6 ± 8.2)	ND (6.96 ± 0.66)	0 (−53.2 ± 14.5)	7.79 ± 1.39 (5.91 ± 0.26)	–30.2 ± 13.9 (−130 ± 15.1)
**21**	ND (6.97 ± 0.13)	0 (−97.2 ± 5.1)	ND (6.57 ± 0.32)	0 (−104.8 ± 7.3)	ND (6.41 ± 0.41)	0 (−64.6 ± 9.4)
**24**	ND (6.42 ± 0.35)	0 (−91.9 ± 15.0)	ND (6.60 ± 0.30)	0 (−88.2 ± 11.7)	7.29 ± 0.86 (4.98 ± 0.37)	–43.4 ± 13.8 (−144 ± 31.9)
**29**	8.07 ± 0.13 (ND)	46.2 ± 2.4 (0)	7.91 ± 0.50 (ND)	26.6 ± 5.4 (−20.2 ± 14.1)	ND (7.17 ± 0.27)	0 (−89.4 ± 7.8)

a The values represent the arithmetic mean ± SEM. ND = cannot be determined.

In parallel, the presence of antagonist effects of the tested compounds was studied using a fixed amount of dopamine (1 μM) at D_4_R (Table 2, pIC_50_ and *I*_max_). Because functionally selective compounds that exert preferential modulation on the G protein or β-arrestin are deemed to be therapeutically useful approaches, β-arrestin2 recruitment assays at D_4_R were also performed to characterize the functional properties of the ligands (Table 2, β-arrestin2 recruitment).

From the data analysis, it emerges that all the para-substituted compounds **18**, **21**, and **24** behave as antagonists toward both G_i_-/G_o_-protein activation and β-arrestin recruitment. On the contrary, 2-pyridyl derivative **29** shows an interestingly biased profile, being a partial agonist with pEC_50_ values similar to those of dopamine toward D_4_R G_i_-/G_o_-protein activation and an antagonist toward β-arrestin recruitment with inhibitory potency and maximal inhibition (*I*_max_) similar to **3**. These results confirm previous findings reporting that ligands with substituents in the para-position behave as antagonists and those with substituents in the ortho-position or bearing a 2-pyridine ring behave as partial agonists.^8^ The functional selectivity of **29** might be exploited to improve the knowledge of the biological functions associated with G-protein activation and β-arrestin recruitment pathways.

### Molecular Modeling Studies

**Figure 3:**
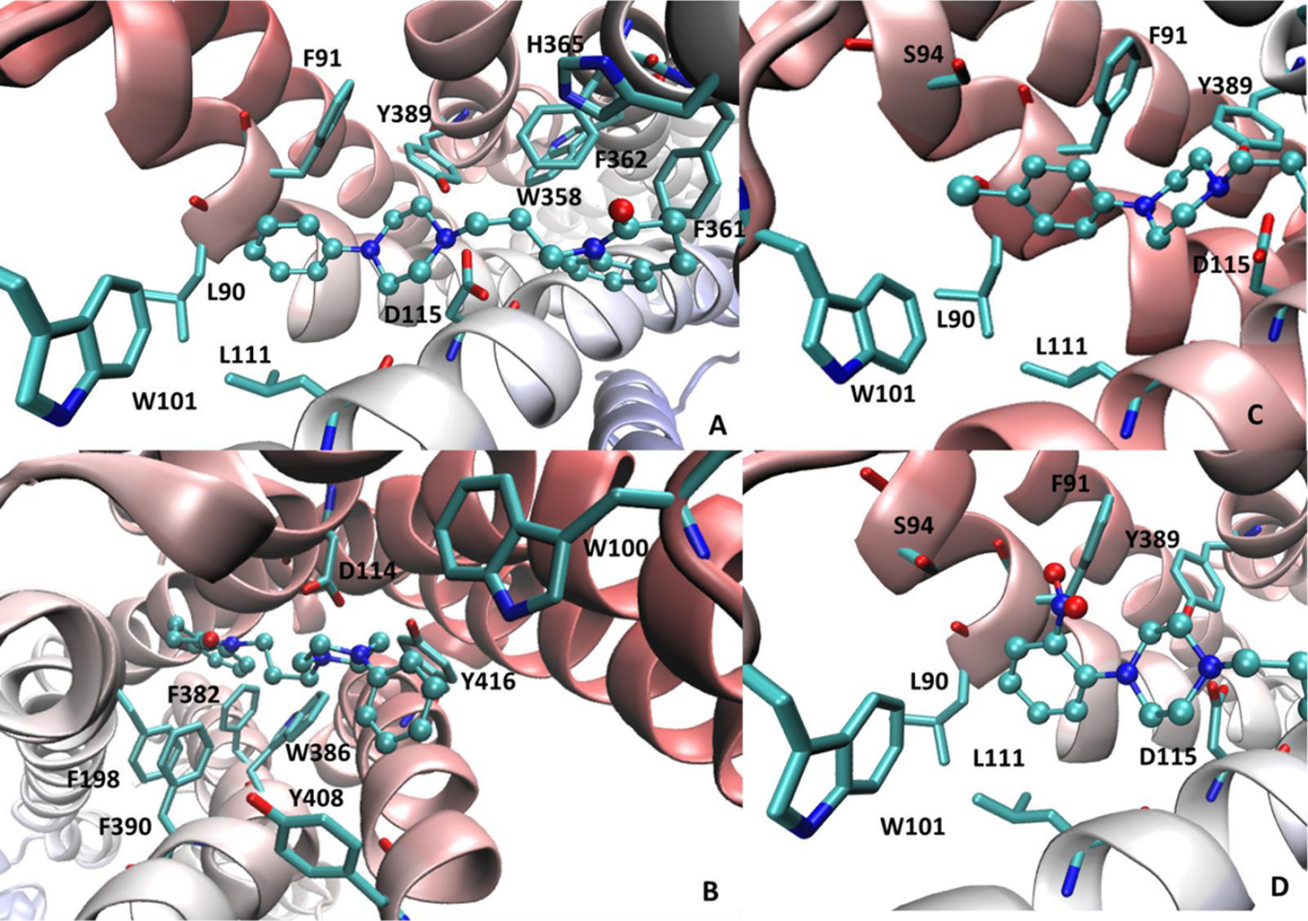
Main interactions stabilizing the putative complexes of **6** within the binding sites of D_4_R (PDB id: 5WIU) (A) and D_2_R (PDB Id: 6CM4) (B). Focus on the interactions engaged by the substituted phenyl ring of **24** (C) and **25** (D) within the D_4_R.

To better rationalize the reported D_4_R affinity values, docking simulations involving the resolved D_4_R structure in complex with nemonapride were performed by using PLANTS. Figure 3A shows the putative complex for **6** and reveals the key ion pair that the protonated piperazine elicits with Asp115 reinforced by the interaction with Tyr389. The quinolinone ring is engaged by a rich set of π–π stacking interactions with the surrounding aromatic residues (e.g., Trp358, Phe361, Phe362, and His365), which can also involve the lactam group. The key role of π–π stacking is confirmed by derivatives **7–11**, in which the quinolinone moiety is replaced by bioisosteric heteroaromatic nuclei. The affinity of these bioisosters is indeed in good agreement with the calculated stacking interactions between heterocycles and aromatic residues (pyrrole > imidazole > tiazole > oxazole).^40^ Quinolinone is also involved in hydrophobic contacts with Leu187 and Val116, and this can explain the good binding affinity of **12**. Lastly, the propyl linker elicits apolar contacts with Met112 and Val193, while the *N*-linked phenyl ring stabilizes π–π stacking with Phe91 and Trp101.

The residues surrounding the phenyl ring can explain the different roles exerted by the added substituents. Specifically, hydrophobic and small substituents (i.e., methyl and chlorine groups) afford a positive contribution regardless of their position because they can always interact with the surrounding apolar residues without inducing steric constraints. As an example, Figure 3C focuses on the arrangement of the para-chloro derivative **24** in which the chlorine atom reinforces the hydrophobic contacts, which also involve Val87, Leu90, and Leu111, and can be engaged by a halogen bond with Ser94. Similar patterns of interactions are seen when the chlorine atom is in meta- or in ortho-positions. In contrast, polar and large substituents can be properly accommodated only in the ortho-position, where they can interact with the Ser94 without exerting steric clashes as exemplified by the ortho-nitro derivative **25** (Figure 3D). In meta- and in para-positions, the added substituents clash against Trp101, as well as against the backbone atoms of Leu90 and Phe91 which closely surround the ligand’s phenyl ring.

With a view to delve into the factors governing the ligand selectivity, similar docking simulations were performed by using the resolved D_2_R structure in complex with the high-affinity D_2_R antagonist risperidone. Figure 3B depicts the putative complex for **6** within the D_2_R binding site and emphasizes some differences with the corresponding complex with D_4_R (Figure 3A) that deserve further attention. The quinolinone ring is completely surrounded by aromatic residues and the more flexible alkyl side chains (as seen in D_4_R) have a marginal impact in this case. On the other side, the *N*-linked phenyl ring is also accommodated within a narrower subpocket (compared to D_4_R), which is lined by Trp100, Phe110, and Tyr408. This can explain why substituents on this ring generally have a detrimental role on the D_2_R affinity unless they can elicit H-bond with Tyr408 or Thr412 (as seen, e.g., with **19**). More generally, the orthosteric D_2_R cavity appears to be smaller and narrower compared to the D_4_R pocket as clearly evidenced by the comparison of their void volumes as computed by FPocket (void volumes equal to 5694 and 4275 Å3 for D_4_R and D_2_R, respectively). This can explain why ligand modifications, that increase the steric hindrance or reduce the flexibility, enhance the D_2_/D_4_ selectivity. The flexibility role is noticeable by considering the positive correlation between the linker length and the D_2_R affinity (as observed for **13**, **14**, and **15**).

Computational analyses were also employed to characterize the ADME/Tox profile of the studied compounds. Thus, Table S2 compiles some relevant physico-chemical descriptors for all the considered compounds. In detail, Table S2 reveals that all compounds show satisfactory physico-chemical profiles (e.g., MW < 500; logP <5; HBA <10; HBD <5; PSA <140 Å^2^; Rotors <10).^41^

The in silico ADME profile of compounds **24** and **29** was further investigated by interrogating the swissADME webserver.^42^ Compound **24** is predicted to be orally bioavailable, brain–blood barrier (BBB) permeant, P-gp substrate with no CYP inhibition apart from CYP2D6. The compound does not violate the most common druglikeness sets of rules (e.g., Lipinski, Ghose, and Veber) without PAINS and Brenk alerts. Its metabolic profile as predicted by the MetaClass method^43^ indicates that **24** can undergo red-ox reactions on nitrogen and Csp2 aromatic atoms. Compound **29** has an ADME profile almost superimposable to that of **24** except for being predicted BBB non-permeant, reasonably due to its lower lipophilicity.

### Biological Studies in GBM Cell Lines

The D_4_R antagonist **24**, showing the highest affinity and selectivity over D_2_R and D_3_R, and the ligand **29,** showing a distinct biased profile, were selected to be evaluated for their potential in affecting the viability of the temozolomide-resistant T98 and temozolomide-sensitive U251 GBM cell lines,^44^ and the primary GBM stem cells GSC#83 as well. In particular, GSC and GBM cell lines were treated with the compounds **24** and **29** (from 5 to 50 μM) for 24h (experimental groups). Parallel cultures (control groups) were incubated for 24 h with temozolomide (Tocris), which is the first-choice chemotherapeutic drug in GBM, the known D_4_R receptor antagonist **1** (Tocris), the D_4_R agonist A412997 (Tocris) (all used at the concentrations ranging from 5 to 50 μM), or the only vehicle.

**Figure 4:**
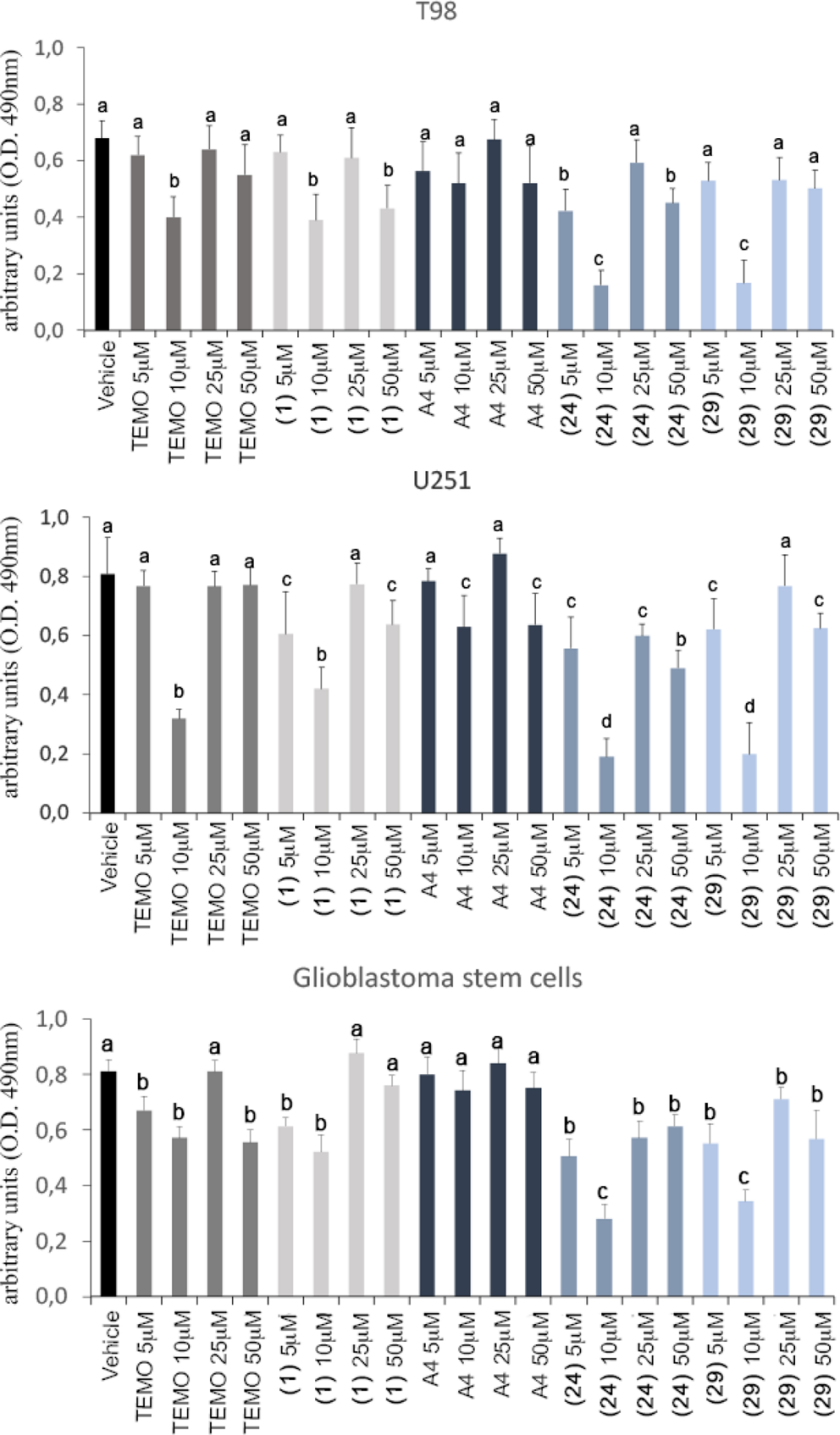
Cell viability assay performed in GBM T98 and U251 cell lines, and in GSC#83. Data were analyzed using two-way analysis of variance. Lowercase letters denote homogeneous subsets (*n* = 6, data shown are means ± standard error, p < 0.05). Vehicle = DMSO. TEMO = temozolomide. A4 = A412997.

Dose–response studies show decreased GBM cell lines and GSC#83’s viability in cultures treated with both compounds **24** and **29**, as well as with controls temozolomide and **1**, with respect to the only vehicle incubated cultures. Conversely, the selective D_4_R agonist A412997 does not significantly modulate cell viability (Figure 4). The maximal efficiency of the compounds, both in the experimental and in the control groups, is reached at a concentration of 10 μM and, more importantly, the treatment with both compounds **24** and **29** induces an increased effect in reducing the T98, U251 cell lines, and GSC#83 viability with respect to the control drugs temozolomide and **1** (Figure 4). Moreover, the results confirm the higher sensitivity of T98 cells versus U251 cells to temozolomide treatment. On the contrary, both the GBM cell lines were equally sensitive in vitro to treatment with D_4_R compounds **24** and **29**.

**Figure 5:**
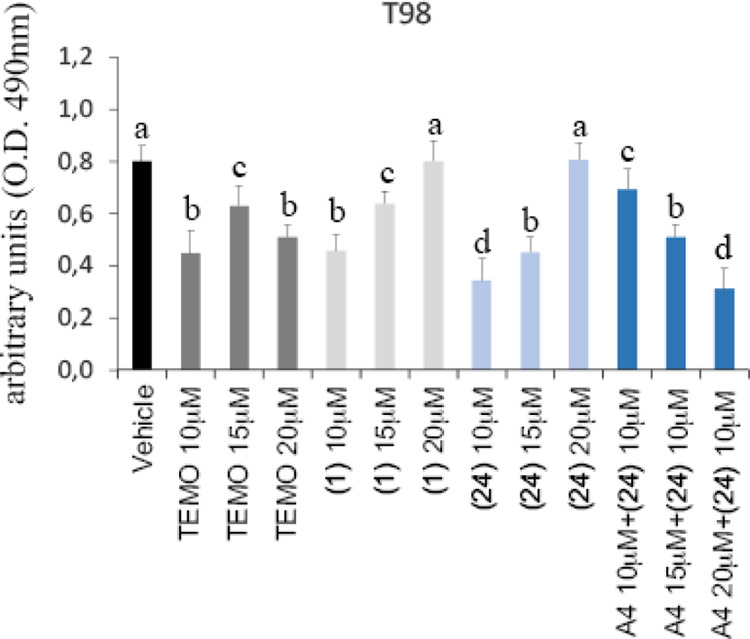
Cell viability assay performed in the T98 cell line. Data were analyzed using two-way analysis of variance. Lowercase letters denote homogeneous subsets (*n* = 6, data shown are means ± standard error, *p* < 0.05). Vehicle = DMSO. TEMO = temozolomide. A4 = A412997.

Because all compounds show maximal antiproliferative activity at a dose of 10 μM, it has been considered of interest to evaluate their activity also in the narrower range of concentration from 10 to 20 μM. Moreover, because all the compounds show a similar activity against the three considered cell lines, the experiment was performed only on T98 cell line. Figure 5 shows that the maximal activity of all the tested compounds is further confirmed at a dose of 10 μM.

The fact that the maximal efficacy was found by using a medium-low dose of effectors tends to exclude the possibility of a non-specific/toxic effect, which usually occurs when high doses of stimulators are used.

Compound **24** was also tested at 10 μM concentration in the presence of increasing concentrations of the D_4_R agonist A412997 (Figure 5). The observation that the agonist at 10 μM contrasts the effect of **24** supports the hypothesis that D_4_R is involved in the antitumor activity of this compound. Analogous to what was observed with **24** and **29**, the effect of A412997 decreases at higher doses (15 and 20 μM).

## Conclusions

Starting from the brain penetrant and D_4_R selective lead compound **6**, in the present study, new ligands endowed with high affinity and selectivity for D_4_R were discovered. In particular, maintaining the *N*-arylpiperazine moiety, the quinolinone portion was replaced by bioisosteric nuclei and the propyl linker by chains of different lengths. Moreover, substituents with different electronic and lipophilic contributions were inserted in ortho-, meta-, and para-positions of the *N*-aryl terminal. SAR studies, supported by molecular modeling simulations, highlighted that the tetrahydroquinolinone nucleus of **6** can be replaced by an *N*-indole or an *N*-tetrahydroquinoline moiety and the propyl linker represents the optimal distance between the lipophilic portion and the basic function. Interestingly, concerning the substitution in the aromatic terminal, the most D_4_R selective compounds were the para-substituted ones, due to the decrease in D_2_R and D_3_R affinity when the substituent is shifted from ortho-to meta- and especially to para-position. From functional studies, while the para-substituted compounds **18**, **21**, and **24** behaved as D_4_R antagonists, the 2-pyridyl derivative **29** showed an interestingly biased profile, being a partial agonist toward D_4_R G_i_-/G_o_-protein activation and an antagonist toward β-arrestin recruitment. In particular, the antagonist **24**, showing the highest affinity and selectivity for D_4_R over D_2_R and D_3_R, and the biased ligand **29** were evaluated for their GBM antitumor activity. They both induced a decreased viability of GBM cell lines and GSC#83, with the maximal efficacy being reached at a concentration of 10 μM. Interestingly, the treatment with both compounds **24** and **29** induces an increased effect in reducing the cell viability with respect to temozolomide, which is the first-choice chemotherapeutic drug in GBM. The observation that the effect of **24** is contrasted by the D_4_R agonist A412997 (10 μM) supports that D_4_R is involved in the antitumor activity of this compound.

Therefore, the new selective D_4_R ligands of the present paper might further shed light on the role played by this subtype in GBM and, especially, become lead compounds for the discovery of new alternatives to the standard treatments such as surgery and radiotherapy, that cannot always be applied, and pharmacological treatments, that are still very limited because of drug resistance.

## Experimental Section

### Chemistry

#### General

Melting points were taken in glass capillary tubes on a Büchi SMP-20 apparatus and are uncorrected. ^1^H NMR spectra were recorded either with a Bruker 500 Ascend (Bruker BioSpin Corporation, Billerica, MA, USA) and Varian Mercury AS400 instruments, and chemical shifts (ppm) are reported relative to tetramethylsilane. Spin multiplicities are given as s (singlet), d (doublet), dd (double doublet), t (triplet), or m (multiplet). IR spectra were recorded on a PerkinElmer 297 instrument and spectral data (not shown because of the lack of unusual features) were obtained for all compounds reported and are consistent with the assigned structures. The microanalyses were recorded on a FLASH 2000 instrument (ThermoFisher Scientific). The elemental composition of the compounds agreed to within ±0.4% of the calculated value. All reactions were monitored by thin-layer chromatography using silica gel plates (60 F254; Merck), visualizing with ultraviolet light. Chromatographic separations were performed on silica gel columns (Kieselgel 40, 0.040–0.063 mm, Merck) by flash chromatography. Compounds were named following IUPAC rules as applied by ChemBioDraw Ultra (version 12.0) software for systematically naming organic chemicals. The purity of the novel compounds was determined by combustion analysis and was ≥95%.

##### 1-(3-(4-Phenylpiperazin-1-yl)propyl)-1H-indole (**7**)

A solution of **36** (1 mmol) in dimethyl formamide (5 mL) was added dropwise to a suspension of sodium hydride (0.04 g, 60% in mineral oil) and dimethyl formamide (5 mL). The resulting mixture was stirred at room temperature for 10 min, followed by the addition of a solution of **35** (1 mmol) in dimethyl formamide (5 mL). The resulting mixture was stirred at 60 °C for 20 h. Then, it was poured onto ice, and the aqueous phase was extracted with EtOAc (2 × 30 mL). The combined organic phases were washed with brine (5 × 30mL) and dried over anhydrous Na_2_SO_4_. The evaporation of the solvent under reduced pressure afforded a residue, which was purified by flash chromatography, eluting with cyclohexane/EtOAc (75:25). Oil was obtained (70% yield). ^1^H NMR (CDCl_3_, 500 MHz): δ 7.62 (d, 1H, *J* = 7.9 Hz), 7.39 (d, 1H, *J* = 8.2 Hz), 7.24–6.87 (m, 8H), 6.50 (d, 1H, *J* = 3.1 Hz), 4.26 (t, 2H, *J* = 6.6 Hz), 3.33–3.20 (m, 4H), 2.74–2.34 (m, 6H), 2.17–2.05 (m, 2H). The free base was transformed into the oxalate salt, which was crystallized from EtOH to give a white solid: mp 99–100 °C, ESI/MS *m*/*z*: 320 [M + H]+, 342 [M + Na]+. Anal. Calcd (C_21_H_25_N_3_.C_2_H_2_O_4_) C, H, N.

##### 1-(3-(4-Phenylpiperazin-1-yl)propyl)-1H-benzo[*d*]imidazole (**8**)

This compound was prepared starting from **37** and **35** following the procedure described for **7**: oil was obtained (42% yield). ^1^H NMR (CDCl_3_, 500 MHz): δ 8.98 (s, 1H), 7.86 (d, 1H, *J* = 7.9 Hz), 7.64 (d, 1H, *J* = 8.0 Hz), 7.47–7.27 (m, 4H), 6.95 (t, 1H, *J* = 7.3 Hz), 6.89 (d, 2H, *J* = 8.1 Hz), 4.64 (t, 2H, *J* = 6.8 Hz), 3.60–3.07 (m, 8H), 2.73–2.64 (m, 2H), 2.25–2.06 (m, 2H). The free base was transformed into the oxalate salt, which was crystallized from EtOH to give a white solid: mp 195–196 °C, ESI/MS *m*/*z*: 321 [M + H]+, 343 [M + Na]+. Anal. Calcd (C_20_H_24_N_4_.C_2_H_2_O_4_) C, H, N.

##### 2-(3-(4-Phenylpiperazin-1-yl)propyl)-1H-benzo[*d*]imidazole (**9**)

Benzene-1,2-diamine (1 mmol) was added to a solution of **38** (1.2 mmol) in 4N HCl in dioxane (10 mL) and the mixture was stirred at reflux for 24 h. The reaction mixture was cooled to room temperature, poured over ice-cold H_2_O (20 mL), neutralized to pH = 7 with NaOH, and extracted with CHCl_3_ (3 × 20 mL). The organic phase was dried over anhydrous Na_2_SO_4_. The evaporation of the solvent under reduced pressure afforded a residue, which was purified by flash chromatography, eluting with EtOAc/MeOH (8:2). A yellow solid was obtained (25% yield). ^1^H NMR (CDCl_3_, 500 MHz): δ 7.59–7.15 (m, 6H), 6.99 (d, 2H, *J* = 7.8 Hz), 6.93 (m, 1H), 3.38–3.32 (m, 4H), 3.17–3.12 (m, 2H), 2.82–2.67 (m, 6H), 2.09–2.04 (m, 3H). The free base was transformed into the oxalate salt, which was crystallized from EtOH to give a white solid: mp 216–219 °C, ESI/MS *m*/*z*: 321 [M + H]+, 343 [M + Na]+. Anal. Calcd (C_20_H_24_N_4_.C_2_H_2_O_4_) C, H, N.

##### 2-(3-(4-Phenylpiperazin-1-yl)propyl)benzo[*d*]oxazole (**10**)

K_2_CO_3_ (5 mmol) and KI (0.2 mmol) were added to a solution of **34** (1 mmol) in DME (10 mL) and the mixture was stirred at room temperature for 10 min, followed by the addition of a solution of **39** (5 mmol) in DME (5 mL). The resulting mixture was stirred at reflux for 15 h. Then, after cooling, EtOAc (20 mL) was added, and the mixture was extracted with brine (3 × 20 mL). The organic phase was dried over anhydrous Na_2_SO_4_. The evaporation of the solvent under reduced pressure afforded a residue, which was purified by flash chromatography, eluting with cyclohexane/EtOAc (7:3). A white solid was obtained (59% yield). ^1^H NMR (CDCl_3_, 500 MHz): δ 7.72–7.26 (m, 6H), 6.96–6.85 (m, 3H), 3.21–3.15 (m, 4H), 3.04 (t, 2H, *J* = 7.5 Hz), 2.66–2.63 (m, 4H), 2.56 (t, 2H, *J* = 7.1 Hz), 2.18–2.12 (m, 2H). The free base was transformed into the oxalate salt, which was crystallized from MeOH to give a white solid: mp 215–216 °C, ESI/MS *m*/*z*: 322 [M + H]^+^, 344 [M + Na]^+^. Anal. Calcd (C_20_H_23_N_3_O.C_2_H_2_O_4_) C, H, N, O.

##### 2-(3-(4-Phenylpiperazin-1-yl)propyl)benzo[*d*]thiazole (**11**)

This compound was prepared starting from **34** and **40** following the procedure described for **10**: oil was obtained (11% yield). ^1^H NMR (CDCl_3_, 500 MHz): δ 7.97 (d, 1H, *J* = 8.1 Hz), 7.86–7.24 (m, 4H), 6.95–6.85 (m, 4H), 3.28–3.18 (m, 6H), 2.75–2.59 (m, 8H). The free base was transformed into the oxalate salt, which was crystallized from EtOH to give a white solid: mp 184–186 °C, ESI/MS *m*/*z*: 338 [M + H]^+^, 360 [M + Na]+. Anal. Calcd (C_20_H_23_N_3_S.C_2_H_2_O_4_) C, H, N, S.

##### 1-(3-(4-Phenylpiperazin-1-yl)propyl)-1,2,3,4-tetrahydroquinoline (**12**)

BH_3_·S(CH_3_)_2_ (0.34 mL) was added to a ice-cooled solution of **6** (1 mmol) in THF (10 mL) at 0 °C under nitrogen, and the mixture was stirred at reflux for 3 h. Then, after cooling to 0 °C, MeOH (10 mL) was added. The mixture was acidified with 2N HCl (5 mL) and stirred at reflux for 1 h. Then, it was cooled to room temperature, basified with 2N NaOH and extracted with CH_2_Cl_2_ (3 × 20 mL). The combined organic phases were dried over anhydrous Na_2_SO_4_.The evaporation of the solvent under reduced pressure afforded a residue, which was purified by flash chromatography, eluting with cyclohexane/EtOAc (7:3). Yellow oil was obtained (59% yield). ^1^H NMR (CDCl_3_, 500 MHz): δ 7.29–6.92 (m, 6H), 6.87 (t, 1H, *J* = 7.3 Hz), 6.62 (d, 1H, *J* = 8.2 Hz), 6.56 (t, 1H, *J* = 7.3 Hz), 3.36–3.22 (m, 8H), 2.79–2.47 (m, 8H), 1.98–1.84 (m, 4H). The free base was transformed into the oxalate salt, which was crystallized from EtOH to give a white solid: m.p. 292–294 °C, ESI/MS *m*/*z*: 336 [M + H]^+^, 358 [M + Na]^+^. Anal. Calcd (C_22_H_29_N_3_.C_2_H_2_O_4_) C, H, N.

##### 1-(2-(4-Phenylpiperazin-1-yl)ethyl)-3,4-dihydroquinolin-2(1H)-one (**13**)

Sodium hydride (0.12 g, 60% in mineral oil) was added to a solution of **41** (10 mmol) in xylene (5 mL), and the mixture was stirred at room temperature for 20 min, followed by the addition of a solution of **42** (5 mmol) in xylene (5 mL). The resulting mixture was stirred at reflux for 4 h. Then, after cooling, it was poured onto ice, and the organic phase was extracted with 5% HCl (3 × 20 mL). The aqueous phase was basified with 2N NaOH and extracted with CH_2_Cl_2_ (3 × 20 mL). The combined organic phases were washed with brine (2 × 20 mL) and dried over anhydrous Na_2_SO_4_. The evaporation of the solvent under reduced pressure afforded a residue, which was purified by flash chromatography, eluting with EtOAc/CH_3_OH (99:1). Oil was obtained (76% yield). ^1^H NMR (CDCl_3_, 400 MHz): δ 7.60–6.95 (m, 9H), 4.04 (m, 2H), 3.74–3.62 (m, 6H), 3.32–3.10 (m, 4H), 2.96 (m, 2H), 2.68 (m, 2H). The free base was transformed into the oxalate salt, which was crystallized from 2-PrOH to give a white solid: mp 210–211 °C, ESI/MS *m*/*z*: 336 [M + H]^+^, 358 [M + Na]^+^. Anal. Calcd (C_21_H_25_N_3_O.C_2_H_2_O_4_) C, H, N.

##### 1-(4-(4-Phenylpiperazin-1-yl)butyl)-3,4-dihydroquinolin-2(1H)-one (**14**)

A solution of **43** (1 mmol) in DMF (5 mL) was added dropwise to a solution of **34** (1 mmol) and K_2_CO_3_ (1.2 mmol) in DMF (10 mL). The reaction mixture was stirred at 70 °C for 4 h; then, it was diluted with water (20 mL) and extracted with EtOAc (2 × 30 mL). The organic layer was washed with brine (5 × 20 mL) and dried over anhydrous Na_2_SO_4_. The evaporation of the solvent under reduced pressure afforded a residue, which was purified by flash chromatography, eluting with EtOAc/CH_3_OH (99:1). Oil was obtained (72% yield). ^1^H NMR (CDCl_3_, 400 MHz): δ 7.39–6.88 (m, 9H), 4.03 (m, 2H), 3.30 (m, 4H), 2.97–2.42 (m. 10H), 1.90–1.55 (m, 4H). The free base was transformed into the oxalate salt, which was crystallized from 2-PrOH to give a white solid: mp 164–165 °C, ESI/MS *m*/*z*: 364 [M + H]^+^, 386 [M + Na]^+^. Anal. Calcd (C_23_H_29_N_3_O.C_2_H_2_O_4_) C, H, N.

##### 1-(5-(4-Phenylpiperazin-1-yl)pentyl)-3,4-dihydroquinolin-2(1H)-one (**15**)

This compound was prepared starting from **44** and **34** following the procedure described for **14**: oil was obtained (51% yield). ^1^HNMR (CDCl_3_, 400 MHz): δ 7.35–6.82 (m, 9H), 3.95 (m, 2H), 3.30 (m, 4H), 2.95 (m, 2H), 2.81–2.45 (m. 8H), 1.85–1.41 (m, 6H). The free base was transformed into the oxalate salt, which was crystallized from 2-PrOH to give a white solid: mp 156–158 °C, ESI/MS *m*/*z*: 378 [M + H]^+^, 400 [M + Na]^+^. Anal. Calcd (C_24_H_31_N_3_O.C_2_H_2_O_4_) C, H, N.

##### 1-(3-(4-(*o*-Tolyl)piperazin-1-yl)propyl)-3,4-dihydroquinolin-2(1H)-one (**16**)

This compound was prepared starting from **45** and **46** following the procedure described for **14**: oil was obtained (47% yield). ^1^H NMR (CDCl_3_): δ 7.31–6.92 (m, 8H), 4.05 (m, 2H), 3.14 (m, 4H), 2.92 (m, 2H), 2.71–2.49 (m, 8H), 2.38 (s, 3H), 1.95 (m, 2H). The free base was transformed into the oxalate salt, which was crystallized from 2-PrOH to give a white solid: mp 149–150 °C, ESI/MS *m*/*z*: 364 [M + H]^+^. Anal. Calcd (C_23_H_29_N_3_O.C_2_H_2_O_4_) C, H, N.

##### 1-(3-(4-(*m*-Tolyl)piperazin-1-yl)propyl)-3,4-dihydroquinolin-2(1H)-one (**17**)

This compound was prepared starting from **45** and **47** following the procedure described for **14**: oil was obtained (55% yield). ^1^H NMR (CDCl_3_, 500 MHz): δ 7.27–6.89 (m, 8H), 4.04 (m, 2H), 3.23 (m, 4H), 2.92 (m, 2H), 2.67 (m, 6H), 2.49 (m, 2H), 2.33 (s, 3H), 1.92 (m, 2H). The free base was transformed into the oxalate salt, which was crystallized from 2-PrOH to give a white solid: mp 185–186 °C, ESI/MS *m*/*z:* 364 [M + H]+. Anal. Calcd (C_23_H_29_N_3_O.C_2_H_2_O_4_) C, H, N.

##### 1-(3-(4-(*p*-Tolyl)piperazin-1-yl)propyl)-3,4-dihydroquinolin-2(1H)-one (**18**)

This compound was prepared starting from **45** and **48** following the procedure described for **14**: oil was obtained (61% yield). ^1^H NMR (CDCl_3_, 500 MHz): δ 7.29–6.85 (m, 8H), 4.04 (m, 2H), 3.18 (m, 4H), 2.93 (dd, 2H, *J* = 18.4 and 11.5 Hz), 2.67 (m, 6H), 2.56 (t, 2H, *J* = 7.1 Hz), 2.29 (s, 3H), 1.92 (m, 2H). The free base was transformed into the oxalate salt, which was crystallized from 2-PrOH to give a white solid: mp 198–199 °C, ESI/MS *m*/*z* 364 [M + H]^+^. Anal. Calcd (C_23_H_29_N_3_O.C_2_H_2_O_4_) C, H, N.

##### 1-(3-(4-(2-Methoxyphenyl)piperazin-1-yl)propyl)-3,4-dihydroquinolin-2(1H)-one (**19**)

This compound was prepared starting from **45** and **49** following the procedure described for **14**: oil was obtained (46% yield). ^1^H NMR (CDCl_3_, 500 MHz): δ 7.31–6.88 (m, 8H), 4.07 (m, 2H), 3.79 (s, 3H), 3.22 (m, 4H), 2.94 (m, 2H), 2.76–2.35 (m, 8H), 1.95 (m, 2H). The free base was transformed into the oxalate salt, which was crystallized from 2-PrOH to give a white solid: mp 164–166 °C, ESI/MS *m*/*z*: 380 [M + H]^+^. Anal. Calcd (C_23_H_29_N_3_O_2_.C_2_H_2_O_4_) C, H, N.

##### 1-(3-(4-(3-Methoxyphenyl)piperazin-1-yl)propyl)-3,4-dihydroquinolin-2(1H)-one (**20**)

This compound was prepared starting from **45** and **50** following the procedure described for **14**: oil was obtained (49% yield). ^1^H NMR (CDCl_3_, 500 MHz): δ 7.26–6.42 (m, 8H), 4.04 (m, 2H), 3.81 (s, 3H), 3.23 (m, 4H), 2.92 (m, 2H), 2.71–2.61 (m, 6H), 2.50 (m, 2H), 1.91 (m, 2H). The free base was transformed into the oxalate salt, which was crystallized from 2-PrOH to give a white solid: mp 153–154 °C, ESI/MS *m*/*z*: 380 [M + H]^+^. Anal. Calcd (C_23_H_29_N_3_O_2_.C_2_H_2_O_4_) C, H, N.

##### 1-(3-(4-(4-Methoxyphenyl)piperazin-1-yl)propyl)-3,4-dihydroquinolin-2(1H)-one (**21**)

This compound was prepared starting from **45** and **51** following the procedure described for **14**: a white solid was obtained (46% yield): mp 98–99 °C. ^1^H NMR (CDCl_3_, 500 MHz): δ 7.25–6.81 (m, 8H), 4.01 (m, 2H), 3.77 (s, 3H), 3.10 (m, 4H), 2.92–2.58 (m, 8H), 2.49 (t, 2H, *J* = 7.2 Hz), 1.90 (m, 2H). The free base was transformed into the oxalate salt, which was crystallized from 2-PrOH to give a white solid: mp 175–177 °C, ESI/MS *m*/*z*: 380 [M + H]+. Anal. Calcd. (C_23_H_29_N_3_O_2_.C_2_H_2_O_4_) C, H, N.

##### 1-(3-(4-(2-Chlorophenyl)piperazin-1-yl)propyl)-3,4-dihydroquinolin-2(1H)-one (**22**)

This compound was prepared starting from **45** and **52** following the procedure described for **14**: oil was obtained (58% yield). ^1^H NMR (CDCl_3_, 500 MHz): δ 7.41–6.92 (m, 8H), 4.07 (m, 2H), 3.19 (m, 4H), 2.94 (m, 2H), 2.73-2-55 (m, 8H), 1.94 (m, 2H). The free base was transformed into the oxalate salt, which was crystallized from EtOH to give a white solid: mp 176–178 °C, ESI/MS *m*/*z*: 384 [M + H]^+^, 406 [M + Na]^+^. Anal. Calcd (C_22_H_26_ClN_3_O.C_2_H_2_O_4_) C, H, N.

##### 1-(3-(4-(3-Chlorophenyl)piperazin-1-yl)propyl)-3,4-dihydroquinolin-2(1H)-one (**23**)

This compound was prepared starting from **45** and **53** following the procedure described for **14**: oil was obtained (61% yield). ^1^H NMR (CDCl_3_, 500 MHz): δ 7.40–6.96 (m, 8H), 4.05 (m, 2H), 3.11 (m, 4H), 2.92 (m, 2H), 2.67 (m, 6H), 2.53 (t, 2H, *J* = 7.1 Hz), 1.91 (m, 2H). The free base was transformed into the oxalate salt, which was crystallized from EtOH to give a white solid: mp 172–173 °C, ESI/MS *m*/*z*: 384 [M + H]+, 406 [M + Na]+. Anal. Calcd (C_22_H_26_ClN_3_O.C_2_H_2_O_4_) C, H, N.

##### 1-(3-(4-(4-Chlorophenyl)piperazin-1-yl)propyl)-3,4-dihydroquinolin-2(1H)-one (**24**)

This compound was prepared starting from **45** and **54** following the procedure described for **14**: oil was obtained (61% yield). ^1^H NMR (CDCl_3_, 500 MHz): δ 7.31–6.83 (m, 8H), 4.04 (m, 2H), 3.19 (m, 4H), 2.93 (dd, 2H, *J* = 19.1 and 12.3 Hz), 2.67 (m, 6H), 2.50 (t, 2H, *J* = 7.0 Hz), 1.90 (m, 2H). The free base was transformed into the oxalate salt, which was crystallized from EtOH to give a white solid: mp 212–214 °C, ESI/MS *m*/*z*: 384 [M + H]^+^, 406 [M + Na]^+^. Anal. Calcd (C_22_H_26_ClN_3_O.C_2_H_2_O_4_) C, H, N.

##### 1-(3-(4-(2-Nitrophenyl)piperazin-1-yl)propyl)-3,4-dihydroquinolin-2(1H)-one (**25**)

This compound was prepared starting from **55** following the procedure described for **14**: oil was obtained (54% yield). ^1^H NMR (CDCl_3_, 500 MHz): δ 7.84–7.00 (m, 8H), 4.07 (m, 2H), 3.12 (m, 4H), 2.94 (m, 2H), 2.63 (m, 6H), 2.52 (m, 2H), 1.97 (m, 2H). The free base was transformed into the oxalate salt, which was crystallized from 2-PrOH to give a yellow solid: mp 172–173 °C, ESI/MS *m*/*z*: 395 [M + H]^+^, 417 [M + Na]^+^. Anal. Calcd (C_22_H_26_N_4_O_3_.C_2_H_2_O_4_) C, H, N.

##### 1-(3-(4-(3-Nitrophenyl)piperazin-1-yl)propyl)-3,4-dihydroquinolin-2(1H)-one (**26**)

This compound was prepared starting from **45** and **56** following the procedure described for **14**: oil was obtained (49% yield). ^1^H NMR (CDCl_3_, 500 MHz): δ 7.84–7.00 (m, 8H), 4.10 (t, 2H, *J* = 7.0 Hz), 3.73 (m, 6H), 3.21 (m, 2H), 2.94 (m, 2H), 2.69 (m, 2H), 2.40 (m, 2H), 1.97 (m, 2H). The free base was transformed into the oxalate salt, which was crystallized from 2-PrOH to give a yellow solid: mp 192–193 °C, ESI/MS *m*/*z*: 395 [M + H]^+^, 417 [M + Na]^+^. Anal. Calcd (C_22_H_26_N_4_O_3_.C_2_H_2_O_4_) C, H, N.

##### 1-(3-(4-(4-Nitrophenyl)piperazin-1-yl)propyl)-3,4-dihydroquinolin-2(1H)-one (**27**)

This compound was prepared starting from **45** and **57** following the procedure described for **14**: oil was obtained (49% yield). ^1^H NMR (CDCl_3_, 500 MHz): δ 8.15 (d, 2H, *J* = 9.5 Hz), 7.28–6.99 (m, 4H), 6.84 (d, 2H, *J* = 9.5 Hz), 4.05 (m, 2H), 3.46 (m, 4H), 2.92 (m, 2H), 2.70–2.62 (m, 6H), 2.51 (t, 2H, *J* = 7.0 Hz), 1.92 (m, 2H). The free base was transformed into the oxalate salt, which was crystallized from 2-PrOH to give a yellow solid: mp 206–207 °C, ESI/MS *m*/*z*: 395 [M + H]^+^, 417 [M + Na]^+^. Anal. Calcd (C_22_H_26_N_4_O_3_.C_2_H_2_O_4_) C, H, N.

##### 2-(4-(3-(2-Oxo-3,4-dihydroquinolin-1(2H)-yl)propyl)piperazin-1-yl)benzonitrile (**28**)

This compound was prepared starting from **45** and **58** following the procedure described for **14**: oil was obtained (67% yield). ^1^H NMR (CDCl_3_, 500 MHz): δ 7.58–7.00 (m, 8H), 4.04 (m, 2H), 3.26 (m, 4H), 2.92 (m, 2H), 2.67 (m, 6H), 2.53 (t, 2H, *J* = 7.1 Hz), 1.90 (m, 2H). The free base was transformed into the oxalate salt, which was crystallized from EtOH to give a pale yellow solid: mp 173–174 °C, ESI/MS *m*/*z* 375 [M + H]^+^, 397 [M + Na]^+^. Anal. Calcd (C_23_H_26_N_4_O.C_2_H_2_O_4_) C, H, N.

##### 1-(3-(4-(Pyridin-2-yl)piperazin-1-yl)propyl)-3,4-dihydroquinolin-2(1H)-one (**29**)

This compound was prepared starting from **45** and **59** following the procedure described for **14**: oil was obtained (48% yield). ^1^H NMR (CDCl_3_, 500 MHz): δ 8.15 (dd, 1H, *J* = 5.0 and 1.5 Hz), 7.46–6.96 (m, 5H), 6.62–6.55 (m, 2H) 3.98 (m, 2H), 3.50–2.90 (m, 6H), 2.65–2.48 (m, 8H), 1.90 (m, 2H). The free base was transformed into the oxalate salt, which was crystallized from 2-PrOH to give a white solid: mp 180–182 °C, ESI/MS *m*/*z*: 351 [M + H]^+^, 373 [M + Na]^+^. Anal. Calcd (C_21_H_26_N_4_O.C_2_H_2_O_4_) C, H, N.

##### 1-(3-(4-(Pyrimidin-2-yl)piperazin-1-yl)propyl)-3,4-dihydroquinolin-2(1H)-one (**30**)

This compound was prepared starting from **45** and **60** following the procedure described for **14**: oil was obtained (62% yield). ^1^H NMR (CDCl_3_, 500 MHz): δ 8.32 (d, 2H, *J* = 4.7 Hz), 7.27–6.99 (m, 4H), 6.50 (t, 1H, *J* = 4.7 Hz), 4.04 (m, 2H), 3.84 (m, 4H), 2.94 (m, 2H), 2.67 (m, 2H), 2.50 (m, 6H), 1.90 (m, 2H). The free base was transformed into the oxalate salt, which was crystallized from 2-PrOH to give a white solid: mp 181–182 °C, ESI/MS *m*/*z*: 352 [M + H]^+^, 374 [M + Na]^+^. Anal. Calcd (C_20_H_25_N_5_O.C_2_H_2_O_4_) C, H, N.

##### 1-(3-(4-(2,3-Dichlorophenyl)piperazin-1-yl)propyl)-3,4-dihydroquinolin-2(1H)-one (**31**)

This compound was prepared starting from **45** and **61** following the procedure described for **14**: oil was obtained (44% yield). ^1^H NMR (CDCl_3_, 500 MHz): δ 7.28–6.93 (m, 7H), 4.02 (m, 2H), 3.09 (m, 4H), 2.89 (dd, 2H, *J* = 16.4 and 9.6 Hz), 2.66 (m, 6H), 2.52 (t, 2H, *J* = 7.2 Hz), 1.90 (m, 2H). The free base was transformed into the oxalate salt, which was crystallized from 2-PrOH to give a white solid: mp 190–191 °C, ESI/MS *m*/*z*: 419 [M + H]^+^. Anal. Calcd (C_22_H_25_Cl_2_N_3_O.C_2_H_2_O_4_) C, H, N.

##### 1-(3-(4-(2,4-Dichlorophenyl)piperazin-1-yl)propyl)-3,4-dihydroquinolin-2(1H)-one (**32**)

This compound was prepared starting from **45** and **62** following the procedure described for **14**: oil was obtained (41% yield). ^1^H NMR (CDCl_3_, 500 MHz): δ 7.39–6.96 (m, 7H), 4.05 (m, 2H), 3.13 (m, 4H), 2.83–2.54 (m, 10H), 1.94 (m, 2H). The free base was transformed into the oxalate salt, which was crystallized from 2-PrOH to give a white solid: mp 209–209 °C, ESI/MS *m*/*z* 419 [M + H]^+^. Anal. Calcd (C_22_H_25_Cl_2_N_3_O.C_2_H_2_O_4_) C, H, N.

##### 1-(3-(4-(3,4-Dichlorophenyl)piperazin-1-yl)propyl)-3,4-dihydroquinolin-2(1H)-one (**33**)

This compound was prepared starting from **45** and **63** following the procedure described for **14**: oil was obtained (41% yield). ^1^H NMR (CDCl_3_, 500 MHz): δ 7.32–6.73 (m, 7H), 4.04 (m, 2H), 3.22 (m, 4H), 2.92 (m, 2H), 2.70–2.44 (m, 8H), 1.91 (m, 2H). The free base was transformed into the oxalate salt, which was crystallized from 2-PrOH to give a white solid: mp 187–189 °C, ESI/MS *m*/*z:* 419 [M + H]^+^. Anal. Calcd (C_22_H_25_Cl_2_N_3_O.C_2_H_2_O_4_) C, H, N.

##### 1-(3-Bromopropyl)-4-phenylpiperazine (**35**)

A solution of **34** (10 mmol) in DMSO (10 mL) was added dropwise to a solution of 1,3-dibromopropane (22 mmol) and KOH (0.6 g) in DMSO (30 mL) and the mixture was stirred for 4 h at 70 °C. Then, it was poured into absolute ethanol to precipitate a solid, which was filtered and rinsed with absolute ethanol three times. Evaporation of the solvent gave **35** as a pale yellow hygroscopic solid (71% yield). ^1^H NMR (CDCl_3_, 500 MHz): δ 7.26 (t, 2H, *J* = 8.7 Hz), 6.93 (d, 2H, *J* = 8.1 Hz), 6.85 (t, 1H, *J* = 6.9 Hz), 3.50 (t, 2H, *J* = 6.6 Hz), 3.20 (m, 4H), 2.62 (m, 4H), 2.55 (t, 2H, *J* = 7.5 Hz), 2.08 (m, 2H).

##### Ethyl 4-(4-Phenylpiperazin-1-yl)butanoate (**38**)

Ethyl 4-bromobutanoate (5.0 mmol) was added to the solution of **34** (5.0 mmol) in ethanol (20 mL) at room temperature and the resulting solution was stirred at reflux for 6 h. After the completion of reaction, the mixture was cooled to room temperature. Sat. NaHCO_3_ (50 mL) was added, and the resulting solution was extracted with CH_2_Cl_2_ (3 × 50 mL). The organic layer was dried over anhydrous Na_2_SO_4_. The evaporation of the solvent under reduced pressure afforded a residue, which was purified by flash chromatography, eluting with CH_2_Cl_2_/CH_3_OH (95:5). Oil was obtained (89% yield). ^1^H NMR (CDCl_3_, 500 MHz): δ 7.30–7.26 (m, 2H), 6.97–6.93 (m, 2H), 6.88 (t, 1H, *J* = 7.3 Hz), 4.18–4.13 (m, 2H), 3.26–3.21 (m, 4H), 2.69–2.63 (m, 4H), 2.49–2.39 (m, 4H), 1.93–1.86 (m, 2H), 1.31–1.26 (m, 3H).

##### 1-(2-Chloroethyl)-4-phenylpiperazine (**42**)

1-Bromo-2-chloroethane 2 (7.2 mmol) was added dropwise to a solution of **34** (6.15 mmol) and K_2_CO_3_ (9.25 mmol) in acetone (10 mL). The reaction mixture was stirred under a nitrogen atmosphere for 15 h. Then, it was filtered, and the filtrate was concentrated under reduced pressure. The residue was diluted with water and extracted with EtOAc (3 × 50 mL). The organic layer was dried over anhydrous Na_2_SO_4_. The evaporation of the solvent under reduced pressure afforded a residue, which was purified by flash chromatography, eluting with cyclohexane/EtOAc (7:3). Oil was obtained (57% yield). ^1^H NMR (CDCl_3_, 400 MHz): δ 7.30–6.80 (m, 5H), 3.63 (t, 2H, *J* = 8.0 Hz), 3.21 (t, 4H), 2.79 (t, 2H, *J* = 8.0 Hz), 2.68 (m, 4H).

##### 1-(4-Bromobutyl)-3,4-dihydroquinolin-2(1H)-one (**43**)

A solution of **41** (13.6 mmol) in DMF (10 mL) was added dropwise to a suspension of sodium hydride (0.54 g, 60% in mineral oil) and DMF (20 mL). The resulting mixture was stirred at room temperature for 20 min, followed by the addition of a solution of 1,4-dibromobutane (13.7 mmol) in DMF (10 mL). The resulting mixture was stirred at room temperature for 20 min. Then, it was poured onto ice, and the aqueous phase was extracted with EtOAc (2 × 30 mL). The combined organic phases were washed with brine (5 × 30mL) and dried over anhydrous Na_2_SO_4_. The evaporation of the solvent under reduced pressure afforded a residue, which was purified by flash chromatography, eluting with cyclohexane/EtOAc (7:3). Oil was obtained (84% yield). ^1^H NMR (CDCl_3_, 400 MHz): δ 7.23–6.96 (m, 4H), 3.93 (m, 2H), 3.40 (m, 2H), 2.85 (t, 2H, *J* = 8 Hz), 2.60 (t, 2H, *J* = 8 Hz), 1.97 (m, 2H), 1.77 (m, 2H).

##### 1-(5-Bromopentyl)-3,4-dihydroquinolin-2(1H)-one (**44**)

This compound was prepared starting from **41** and 1,5-dibromopentane following the procedure described for **44**: oil was obtained (89% yield). ^1^H NMR (CDCl_3_, 400 MHz): δ 6.96–7.23 (m, 4H), 3.95 (m, 2H), 3.40 (m, 2H), 2.91 (t, 2H, *J* = 7.8 Hz), 2.63 (t, 2H, *J* = 7.8 Hz), 1.94 (m, 2H), 1.68 (m, 2H), 1.52 (m, 2H).

##### 1-(3-Bromopropyl)-3,4-dihydroquinolin-2(1H)-one (**45**)

This compound was prepared starting from **41** and 1,3-dibromopropane following the procedure described for **44**: oil was obtained (56% yield). ^1^H NMR (CDCl_3_, 400 MHz): δ 7.30–6.99 (m, 4H), 4.11 (m, 2H), 3.50 (t, 2H, *J* = 6.5 Hz), 2.93 (m, 2H), 2.67 (dd, 2H, *J* = 8.5 and 6.6 Hz), 2.26 (m, 2H).

### D_2_-like Radioligand Binding Assays

Membranes were prepared from HEK293 cells stably expressing human D_2L_, D_3_, or D_4.4_, grown in a 50:50 mix of DMEM and Ham’s F12 culture media, supplemented with 20 mM HEPES, 2 mM l-glutamine, 0.1 mM non-essential amino acids, 1X antibiotic/antimycotic, 10% heat-inactivated fetal bovine serum, and 200 μg/mL hygromycin (Life Technologies, Grand Island, NY) and kept in an incubator at 37 °C and 5% CO_2_. Upon reaching 80–90% confluence, cells were harvested using pre-mixed Earle’s balanced salt solution (EBSS) with 5 mM EDTA (Life Technologies) and centrifuged at 3,000 rpm for 10 min at 21 °C. The supernatant was removed, and the pellet was resuspended in 10 ml hypotonic lysis buffer (5 mM MgCl_2_, 5 mM Tris, pH 7.4 at 4 °C) and centrifuged at 20,000 rpm for 30 min at 4 °C. The pellet was then resuspended in the respective fresh binding buffers made from 8.7 g/L Earle’s Balanced Salts without phenol red (US Biological, Salem, MA), 2.2 g/L sodium bicarbonate, pH to 7.4 for the [^3^H]*N*-methylspiperone assay, or 50 mM Tris, 10 mM MgCl_2_, 1 mM EDTA, pH to 7.4 for the [^3^H]-(*R*)-(+)-7-OH-DPAT assay. A Bradford protein assay (Bio-Rad, Hercules, CA) was used to determine the protein concentration, and membranes were either stored at −80 °C for later use (500 μg/ml for [^3^H]*N*-methylspiperone assay), or used fresh {∼500–600 μg/ml for [^3^H]-(*R*)-(+)-7-OH-DPAT assay}.

Radioligand competition binding experiments were conducted as previously described.^36,37^ Test compounds were freshly dissolved in 30% DMSO and 70% H_2_O to a stock concentration of 10 mM. Each test compound was then diluted into 10 half-log serial dilutions using a 30% DMSO vehicle. For the [^3^H]*N*-methylspiperone assay, previously frozen membranes were diluted in fresh EBSS to a 200 μg/mL (for D_2_ or D_3_) or 300 μg/mL (D_4_) stock for binding. Radioligand competition experiments were conducted in 96-well plates containing 300 mL fresh binding buffer, 50 mL of diluted test compound, 100 mL of membranes ([^3^H]*N*-methylspiperone: 20 μg/well total protein for D_2_ or D_3_, 30 μg/well total protein for D_4;_ [^3^H]-(*R*)-(+)-7-OH-DPAT: ∼50–60 μg/well total protein concentration for D_4_), and 50 mL of [^3^H]*N*-methylspiperone (0.4 nM final concentration; Novandi Chemistry, SE) or [^3^H]-(*R*)-(+)-7-OH-DPAT (3 nM final concentration, Perkin Elmer), diluted in their respective binding buffer. Nonspecific binding was determined using 10 μM (+)-butaclamol (Sigma-Aldrich, St. Louis, MO) and total binding was determined with 30% DMSO vehicle (3% final concentration in the wells). All compound dilutions were tested in triplicate and the reaction was incubated for 1 h ([^3^H]*N*-methylspiperone) or 1.5 h ([^3^H]-(*R*)-(+)-7-OH-DPAT), at room temperature. The reaction was terminated by filtration through a PerkinElmer Uni-Filter-96 GF/B or GF/C, presoaked in 0.5% polyethylenimine for all the incubation time, using a Brandel 96-well plates Harvester manifold (Brandel Instruments, Gaithersburg, MD). The filters were washed 3 times with 3 mL (3 × 1 mL/well) of ice-cold binding buffer. Then, 65 μL of PerkinElmer MicroScint 20 scintillation cocktail was added to each well, and filters were counted using a PerkinElmer MicroBeta microplate counter. The counter efficiency was experimentally determined for each radioligands, and aliquots of the radioligand dilutions were measured to quantify the exact amount of [^3^H] ligand added in each experiment. IC_50_ values for each compound were determined from dose–response curves, and K_i_ values were calculated using the Cheng–Prusoff equation. When a complete inhibition could not be achieved at the highest tested concentrations, *K*_i_ values have been extrapolated by constraining the bottom of the dose–response curves (=0% residual specific binding) in the nonlinear regression analysis. *K*_d_ values for both radioligands were determined via separate homologous competitive binding experiments. These analyses were performed using GraphPad Prism version 9.00 for Macintosh (GraphPad Software, San Diego, CA). *K*_i_ values were determined from at least three independent experiments and are reported as mean ± SEM.

### BRET Assays

To perform BRET functional assays, human embryonic kidney cells 293T (HEK-293T) were transfected with constructs that include the donor enzyme RLuc8 (renilla luciferase variant) and the acceptor protein mVenus (yellow fluorescent variant) as a BRET pair fused to the respective proteins under study. In the G protein activation assays, the Gαi1 or GαoA subunit was fused to RLuc8 and the Gγ2 subunit to the mVenus. For the recruitment assays, β-arrestin was fused to mVenus and the D4 receptor was fused to RLuc8, as previously described.^45^ HEK293T cells were grown on 10 cm dishes in the DMEM culture medium supplemented with 10% fetal bovine serum (FBS), 2 mM glutamine and 1% penicillin–streptomycin and transiently transfected with 15 μg total plasmid cDNA using 30 μg polyethyleneimine (Sigma-Aldrich) as a transfection agent with 6 h incubation terminated by the medium change. After 48 h, the transfected cells were washed, harvested, and resuspended in 1X PBS containing 0.1% glucose and 200 μM Na bisulfite. Approximately, 2 × 105 cells/well were distributed into 96-well plates (White Lumitrac 200, Greiner bio-one, Monroe, NC, USA) and 5 μM of the luciferase substrate, coelenterazine H, was added to each well. After 2min, the ligands were also transferred to each well. Antagonists were preincubated with the cells 10 min prior to the addition of ligands. Luminescence was measured at the RLuc8 wavelength (485 nm) and fluorescence at the m-Venus wavelength window (530nm), 2.5 min after ligands were added, using a PherastarFSX plate reader (BMG Labtech, Cary, NC, USA). The BRET ratio was expressed as the ratio of fluorescence and luminescence and the background determined in cells expressing RLuc8 alone was subtracted to obtain net BRET values. Generation of dose–response curves represented in drug-induced BRET ratios in response to the respective drugs as well as statistical analysis were performed using Prism 9 (GraphPad Software, San Diego, CA, USA).

### Experimental Details of Modeling Studies

Docking simulations involved the resolved D_4_R structure in complex with nemonapride (PDB Id: 5WIU) as well as the resolved D_2_R structure in complex with risperidone (PDB Id: 6CM4). The protein structures were prepared as previously described.^36,37^ The ligands were simulated in their protonated state and their 3D structure was optimized by using the VEGA suite of programs.^46^ Docking simulations were performed by using PLANTS^47^ and focusing the searches within a 10 Å radius sphere around the co-crystallized ligand. For each molecule, 10 poses were generated by using the ChemPLP scoring functions and the speed parameter equal to 1. The so computed complexes were finally minimized and analyzed using ReScore+0.^48^ Authors will release the atomic coordinates upon article publication.

### Experimental Details of Biological Studies in GBM Cell Lines

#### GBM Cell Lines and GSC Cultures

GBM cell lines T98 and U251 (grade IV) were obtained as previously described.^49^ Cells were grown until 80% of confluence in Eagle’s minimum essential medium (EMEM, Sigma, Merck Life Science S.r.l. Milano, Italy) plus 10% heat-inactivated fetal calf serum (HIFCS, Life Technologies, Monza, Italy), penicillin (100 U/mL), and streptomycin (50 μg/mL) in a humidified atmosphere of 5% CO_2_ at 37 °C. GSC#83 line previously characterized by Ricci-Vitiani et al.^50^ was isolated from a surgical sample of adult patients with a primitive brain tumor undergoing partial surgical resection at the Institute of Neurosurgery, Catholic University School of Medicine, in Rome, Italy. Patients were eligible for the study if a diagnosis of glioblastoma multiforme was established histologically according to the WHO classification.^51^ Informed consent was obtained before surgery according to the Ethical Committee of Catholic University School of Medicine. GSC culture was established from the tumor specimen through mechanical dissociation and culturing in DMEM/F12 serum-free medium containing 2 mM glutamine, 0.6% glucose, 9.6 g/mL putrescine, 6.3 ng/mL progesterone, 5.2 ng/mL sodium selenite, 0.025 mg/mL insulin, and 0.1 mg/mL transferrin sodium salt (Sigma-Aldrich, St. Louis, MO, USA), supplemented with EGF and bFGF. GSC line grown as floating spheres in serum-free medium supplemented with mitogens showed an undifferentiated state, as indicated by their rounded morphology, high nuclear/cytoplasm ratio. Human GSC#83 line was authenticated by short tandem repeat (STR) profiling according to the American National Standards Institute/American Type Culture Collection Standard ASN-0002-2011.12 using the Cell line Integrated Molecular Authentication database (CLIMA),13 and Cellosaurus STR database (CLASTR) of the Cellosaurus database (ExPASy) at the IRCC Ospedale Policlinico San Martino, Interlab Cell Line Collection (ICLC), Biological Resource Center (CRB-HSM), Genova, Italy.^52^

### MTS Assay

T98 and U251 cell lines as well as GSC#83 were plated on 96 well culture plate at a density of 5,000 cells/well and grown as above described until 80% of confluence. Then, cells were treated with the following compounds: **24** and **29** at different concentrations starting from 5 μM to 50 μM diluted in DMSO (Sigma, Milano, Italy) for 24 h. Controls were performed by incubating the cultures for 24 h with different doses (ranging from 5 to 50 μM) of temozolomide, the D_4_R antagonists **1**, the D_4_R agonist A412997, and with the only vehicle (DMSO). The next steps were performed as previously described.^53^ Briefly, cultures were incubated with 200 μL/well of CellTiter 96 Aqueous One Solution Reagent (Promega Italia srl, Milano, Italy) and the colored formazan product was measured by reading the absorbance at 490 nm using a 96-well plate reader (Tecan infinite multiplate reader).

### Statistical Analysis

All the data were expressed as a mean ± standard error (s.e). Two-way analysis of variance (ANOVA) was used to compare the variables. The Tukey test was used in multiple comparisons among all groups. All the statistical analyses were performed using the GraphPad Prism (v 6.01) on a personal computer O.S. Windows 10. Data were presented as mean ± s.e. Values of *P* < 0.05 were considered significant.

## Supporting Information Available

Elemental analysis results for compounds **7**–**33**, relevant physico-chemical descriptors for compounds **5-33**, and HPLC chromatograms and experimental details for **24** and **29** (PDF)
Molecular formula strings (CSV)

## Abbreviations USED

AC: adenylyl cyclase
BBB: brain–blood barrier
BRET: bioluminescence resonance energy transfer
cAMP: adenosine 3′,5′-cyclic monophosphate
CNS: central nervous system
DA: dopamine
DR: dopamine receptors
GBM: glioblastoma
GPCR: G protein-coupled receptor
ICL_3_: third intracellular loop
TM: transmembrane

## References

[ref1] Missale C.; Nash S. R.; Robinson S. W.; Jaber M.; Caron M. G. Dopamine Receptors: From Structure to Function. Physiol. Rev. 1998, 78, 189–225. 10.1152/physrev.1998.78.1.189. 9457173

[ref2] Beaulieu J. M.; Gainetdinov R. R. The Physiology, Signaling, and Pharmacology of Dopamine Receptors. Pharmacol. Rev. 2011, 63, 182–217. 10.1124/pr.110.002642. 21303898

[ref3] Martel J. C.; Gatti McArthur S. Dopamine Receptor Subtypes, Physiology and Pharmacology: New Ligands and Concepts in Schizophrenia. Front. Pharmacol. 2020, 11, 1003 10.3389/fphar.2020.01003. 32765257 PMC7379027

[ref4] Beaulieu J. M.; Espinoza S.; Gainetdinov R. R. Dopamine Receptors - Iuphar Review 13. Br. J. Pharmacol. 2015, 172, 1–23. 10.1111/bph.12906. 25671228 PMC4280963

[ref5] Xin J.; Fan T.; Guo P.; Wang J. Identification of Functional Divergence Sites in Dopamine Receptors of Vertebrates. Comput. Biol. Chem. 2019, 83, 107140 10.1016/j.compbiolchem.2019.107140. 31715491

[ref6] Vallone D.; Picetti R.; Borrelli E. Structure and Function of Dopamine Receptors. Neurosci. Biobehav. Rev. 2000, 24, 125–132. 10.1016/s0149-7634(99)00063-9. 10654668

[ref7] Huff R. M.; Chio C. L.; Lajiness M. E.; Goodman L. V. Signal Transduction Pathways Modulated by D2-Like Dopamine Receptors. Adv. Pharmacol. 1998, 42, 454 10.1016/s1054-3589(08)60786-3. 9327937

[ref8] Giorgioni G.; Del Bello F.; Pavletic P.; Quaglia W.; Botticelli L.; Cifani C.; Micioni Di Bonaventura E.; Micioni Di Bonaventura M. V.; Piergentili A. Recent Findings Leading to the Discovery of Selective Dopamine D_4_ Receptor Ligands for the Treatment of Widespread Diseases. Eur. J. Med. Chem. 2021, 212, 113141 10.1016/j.ejmech.2020.113141. 33422983

[ref9] Botticelli L.; Micioni Di Bonaventura E.; Del Bello F.; Giorgioni G.; Piergentili A.; Romano A.; Quaglia W.; Cifani C.; Micioni Di Bonaventura M. V. Underlying Susceptibility to Eating Disorders and Drug Abuse: Genetic and Pharmacological Aspects of Dopamine D4 Receptors. Nutrients 2020, 12, 2288 10.3390/nu12082288. PMC7468707 32751662

[ref10] Lindsley C. W.; Hopkins C. R. Return of D_4_ Dopamine Receptor Antagonists in Drug Discovery. J. Med. Chem. 2017, 60, 7233–7243. 10.1021/acs.jmedchem.7b00151. 28489950

[ref11] Tol H. H.; Wu C. M.; Guan H. C.; Ohara K.; Bunzow J. R.; Civelli O.; Kennedy J.; Seeman P.; Niznik H. B.; Jovanovic V. Multiple Dopamine D4 Receptor Variants in the Human Population. Nature 1992, 358, 149–152. 10.1038/358149a0. 1319557

[ref12] Asghari V.; Sanyal S.; Buchwaldt S.; Paterson A.; Jovanovic V.; Van Tol H. H. Modulation of Intracellular Cyclic AMP Levels by Different Human Dopamine D4 Receptor Variants. J. Neurochem. 1995, 65, 1157 10.1046/j.1471-4159.1995.65031157.x. 7643093

[ref13] Valerio A.; Belloni M.; Gorno M. L.; Tinti C.; Memo M.; Spano P. Dopamine D_2_, D_3_, and D_4_ Receptor mRNA Levels in Rat Brain and Pituitary During Aging. Neurobiol. Aging 1994, 15, 713–719. 10.1016/0197-4580(94)90053-1. 7891826

[ref14] Ariano M. A.; Wang J.; Noblett K. L.; Larson E. R.; Sibley D. R. Cellular Distribution of the Rat D_4_ Dopamine Receptor Protein in the CNS Using Anti-Receptor Antisera. Brain Res. 1997, 752, 26–34. 10.1016/s0006-8993(96)01422-9. 9106437

[ref15] Jaber M.; Robinson S. W.; Missale C.; Caron M. G. Dopamine Receptors and Brain Function. Neuropharmacology 1996, 35, 1503–1519. 10.1016/s0028-3908(96)00100-1. 9025098

[ref16] Rosas-Cruz A.; Salinas-Jazmín N.; Velázquez M. A. V.-. Dopamine Receptors in Cancer: Are They Valid Therapeutic Targets?. Technol. Cancer Res. Treat. 2021, 20, 1–13. 10.1177/15330338211027913. PMC8255587 34212819

[ref17] Dolma S.; Selvadurai H. J.; Lan X.; Lee L.; Kushida M.; Voisin V.; Whetstone H.; So M.; Aviv T.; Park N.; Zhu X.; Xu C.; Head R.; Rowland K. J.; Bernstein M.; Clarke I. D.; Bader G.; Harrington L.; Brumell J. H.; Tyers M.; Dirks P. B. Inhibition of Dopamine Receptor D4 Impedes Autophagic Flux, Proliferation, and Survival of Glioblastoma Stem Cells. Cancer Cell 2016, 29, 859–873. 10.1016/j.ccell.2016.05.002. 27300435 PMC5968455

[ref18] Zhou Y.; Cao C.; He L.; Wang X.; Zhang X. C. Crystal Structure of Dopamine Receptor D4 Bound to the Subtype Selective Ligand, L745870. ELife 2019, 8, e48822 10.7554/eLife.48822. 31750832 PMC6872212

[ref19] Wang S.; Wacker D.; Levit A.; Che T.; Betz R. M.; McCorvy J. D.; Venkatakrishnan A. J.; Huang X. P.; Dror R. O.; Shoichet B. K.; Roth B. L. D_4_ Dopamine Receptor High-Resolution Structures Enable the Discovery of Selective Agonists. Science 2017, 358, 381–386. 10.1126/science.aan5468. 29051383 PMC5856174

[ref20] Bonifazi A.; Yano H.; Del Bello F.; Farande A.; Quaglia W.; Petrelli R.; Matucci R.; Nesi M.; Vistoli G.; Ferre S.; Piergentili A. Synthesis and Biological Evaluation of a Novel Series of Heterobivalent Muscarinic Ligands Based on Xanomeline and 1-[3-(4-Butylpiperidin-1-yl)propyl]-1,2,3,4-tetrahydroquinolin-2-one (77-LH-28-1). J. Med. Chem. 2014, 57, 9065–9077. 10.1021/jm501173q. 25275964

[ref21] Del Bello F.; Bonifazi A.; Giorgioni G.; Cifani C.; Micioni Di Bonaventura M. V.; Petrelli R.; Piergentili A.; Fontana S.; Mammoli V.; Yano H.; Matucci R.; Vistoli G.; Quaglia W. 1-[3-(4-Butylpiperidin-1-yl)propyl]-1,2,3,4-tetrahydroquinolin-2-one (77-LH-28-1) as a Model for the Rational Design of a Novel Class of Brain Penetrant Ligands with High Affinity and Selectivity for Dopamine D_4_ Receptor. J. Med. Chem. 2018, 61, 3712–3725. 10.1021/acs.jmedchem.8b00265. 29589445

[ref22] Del Bello F.; Bonifazi A.; Giannella M.; Giorgioni G.; Piergentili A.; Petrelli R.; Cifani C.; Micioni Di Bonaventura M. V.; Keck T. M.; Mazzolari A.; Vistoli G.; Cilia A.; Poggesi E.; Matucci R.; Quaglia W. The Replacement of the 2-Methoxy Substituent of N-((6,6-diphenyl-1,4-dioxan-2-yl)methyl)-2-(2-methoxyphenoxy)ethan-1-amine Improves the Selectivity for 5-HT_1A_ Receptor over α_1_-Adrenoceptor and D_2_-Like Receptor Subtypes. Eur. J. Med. Chem. 2017, 125, 233–244. 10.1016/j.ejmech.2016.09.026. 27662034

[ref23] Yarim M.; Koksal M.; Schepmann D.; Wünsch B. Synthesis and in Vitro Evaluation of Novel Indole-Based Sigma Receptors Ligands. Chem. Biol. Drug Des. 2011, 78, 869–875. 10.1111/j.1747-0285.2011.01215.x. 21848665

[ref24] Yan Z.; Lirong Z.; Jie Z.; Xin Z.; Peng W. Benzo Five-Membered Nitrogen Heterocyclic Piperidine or Piperazine Derivatives and Preparation Methods and Pharmaceutical Compositions Thereof. U.S. Patent 9,802,929 B2, 2015.

[ref25] Mokrosz J. L.; Duszyńska B.; Paluchowska M. H. Structure-Activity Relationship Studies of CNS Agents, XV: N-[omega-(4-aryl-1-piperazinyl)alkyl]-2-oxo-1,2,3,4-tetrahydroquinolines and -4-oxo-1,2,3,4-tetrahydropyrazino[1,2-a]indoles: New, Highly Potent 5-HT_1A_ Ligands. Arch. Pharm. 1994, 327, 529–531. 10.1002/ardp.19943270811. 7944908

[ref26] López L.; Selent J.; Ortega R.; Masaguer C. F.; Domínguez E.; Areias F.; Brea J.; Loza M. I.; Sanz F.; Pastor M. Synthesis, 3D-QSAR, and Structural Modeling of Benzolactam Derivatives with Binding Affinity for the D_2_ and D_3_ Receptors. ChemMedChem 2010, 5, 1300–1317. 10.1002/cmdc.201000101. 20544783

[ref27] Oshiro Y.; Sakurai Y.; Sato S.; Kurahashi N.; Tanaka T.; Kikuchi T.; Tottori K.; Uwahodo Y.; Miwa T.; Nishi T. 3,4-Dihydro-2(1H)-quinolinone as a Novel Antidepressant Drug: Synthesis and Pharmacology of 1-[3-[4-(3-Chlorophenyl)-1-piperazinyl]propyl]-3,4- dihydro-5-methoxy-2(1H)-quinolinone and Its Derivatives. J. Med. Chem. 2000, 43, 177–189. 10.1021/jm980333v. 10649973

[ref28] Santos M. A.; Marques S. M.; Tuccinardi T.; Carelli P.; Panelli L.; Rossello A. Design, Synthesis and Molecular Modeling Study of Iminodiacetyl Monohydroxamic Acid Derivatives as MMP Inhibitors. Bioorg. Med. Chem. 2006, 14, 7539 10.1016/j.bmc.2006.07.011. 16875829

[ref29] Na Y. H.; Hong S. H.; Lee J. H.; Park W. K.; Baek D. J.; Koh H. Y.; Cho Y. S.; Choo H.; Pae A. N. Novel Quinazolinone Derivatives as 5-HT_7_ Receptor Ligands. Bioorg. Med. Chem. 2008, 16, 2570–2578. 10.1016/j.bmc.2007.11.049. 18083580

[ref30] Sampson D.; Zhu X. Y.; Eyunni S. V.; Etukala J. R.; Ofori E.; Bricker B.; Lamango N. S.; Setola V.; Roth B. L.; Ablordeppey S. Y. Identification of a New Selective Dopamine D_4_ Receptor Ligand. Bioorg. Med. Chem. 2014, 22, 3105–3114. 10.1016/j.bmc.2014.04.026. 24800940 PMC4096627

[ref31] Zhu X. Y.; Etukala J. R.; Eyunni S. V. K.; Setola V.; Roth B. L.; Ablordeppey S. Y. Benzothiazoles as Probes for the 5-HT_1A_ Receptor and the Serotonin Transporter (SERT): A Search for New Dual-Acting Agents as Potential Antidepressants. Eur. J. Med. Chem. 2012, 53, 124–132. 10.1016/j.ejmech.2012.03.042. 22520153 PMC3361616

[ref32] Zhou B.; Hong K. H.; Ji M.; Cai J. Design, Synthesis, and Biological Evaluation of Structurally Constrained Hybrid Analogues Containing Ropinirole Moiety as a Novel Class of Potent and Selective Dopamine D3 Receptor Ligands. Chem. Biol. Drug Des. 2018, 92, 1597–1609. 10.1111/cbdd.13324. 29710404

[ref33] Zampieri D.; Vio L.; Fermeglia M.; Pricl S.; Wünsch B.; Schepmann D.; Romano M.; Mamolo M. G.; Laurini E. Computer-Assisted Design, Synthesis, Binding and Cytotoxicity Assessments of New 1-(4-(Aryl(methyl)amino)butyl)-heterocyclic Sigma 1 Ligands. Eur. J. Med. Chem. 2016, 121, 712–726. 10.1016/j.ejmech.2016.06.001. 27366902

[ref34] Felding J.; Bang-Andersen B.; Smith G.; Paul; Andersen K. Indole Derivatives Useful for the Treatment of CNS Disorders, ZA,200,209,958 B, 2002.

[ref35] Sams A. G.; Hentzer M.; Mikkelsen G. K.; Larsen K.; Bundgaard C.; Plath N.; Christoffersen C. T.; Bang-Andersen B. Discovery of N-{1-[3-(3-oxo-2,3-dihydrobenzo[1,4]oxazin-4-yl)propyl]piperidin-4-yl}-2-phenylacetamide (Lu AE51090): An Allosteric Muscarinic M_1_ Receptor Agonist with Unprecedented Selectivity and Procognitive Potential. J. Med. Chem. 2010, 53, 6386–6397. 10.1021/jm100697g. 20684563

[ref36] Bonifazi A.; Newman A. H.; Keck T. M.; Gervasoni S.; Vistoli G.; Del Bello F.; Giorgioni G.; Pavletic P.; Quaglia W.; Piergentili A. Scaffold Hybridization Strategy Leads to the Discovery of Dopamine D_3_ Receptor-Selective or Multitarget Bitopic Ligands Potentially Useful for Central Nervous System Disorders. ACS Chem. Neurosci. 2021, 12, 3638–3649. 10.1021/acschemneuro.1c00368. 34529404 PMC8498988

[ref37] Del Bello F.; Ambrosini D.; Bonifazi A.; Newman A. H.; Keck T. M.; Giannella M.; Giorgioni G.; Piergentili A.; Cappellacci L.; Cilia A.; Franchini S.; Quaglia W. Multitarget 1,4-Dioxane Compounds Combining Favorable D_2_-Like and 5-HT_1A_ Receptor Interactions with Potential for the Treatment of Parkinson’s Disease or Schizophrenia. ACS Chem. Neurosci. 2019, 10, 2222–2228. 10.1021/acschemneuro.8b00677. 30609891 PMC8378419

[ref38] Stewart A. O.; Cowart M. D.; Moreland R. B.; Latshaw S. P.; Matulenko M. A.; Bhatia P. A.; Wang X.; Daanen J. F.; Nelson S. L.; Terranova M. A.; Namovic M. T.; Donnelly-Roberts D. L.; Miller L. N.; Nakane M.; Sullivan J. P.; Brioni J. D. Dopamine D4 Ligands and Models of Receptor Activation: 2-(4-Pyridin-2-ylpiperazin-1-ylmethyl)-1H-benzimidazole and Related Heteroarylmethylarylpiperazines Exhibit a Substituent Effect Responsible for Additional Efficacy Tuning. J. Med. Chem. 2004, 47, 2348–2355. 10.1021/jm0305669. 15084133

[ref39] Keck T. M.; Free R. B.; Day M. M.; Brown S. L.; Maddaluna M. S.; Fountain G.; Cooper C.; Fallon B.; Holmes M.; Stang C. T.; Burkhardt R.; Bonifazi A.; Ellenberger M. P.; Newman A. H.; Sibley D. R.; Wu C.; Boateng C. A. Dopamine D4 Receptor-Selective Compounds Reveal Structure–Activity Relationships That Engender Agonist Efficacy. J. Med. Chem. 2019, 62, 3722–3740. 10.1021/acs.jmedchem.9b00231. 30883109 PMC6466480

[ref40] Bootsma A. N.; Doney A. C.; Wheeler S. E. Predicting the Strength of Stacking Interactions between Heterocycles and Aromatic Amino Acid Side Chains. J. Am. Chem. Soc. 2019, 141, 11027–11035. 10.1021/jacs.9b00936. 31267750

[ref41] Matsson P.; Doak B. C.; Over B.; Kihlberg J. Cell Permeability Beyond the Rule of 5. Adv. Drug Del. Rev. 2016, 101, 42–61. 10.1016/j.addr.2016.03.013. 27067608

[ref42] Daina A.; Michielin O.; Zoete V. Swissadme: A Free Web Tool to Evaluate Pharmacokinetics, Drug-Likeness and Medicinal Chemistry Friendliness of Small Molecules. Sci. Rep. 2017, 7, 42717 10.1038/srep42717. 28256516 PMC5335600

[ref43] Mazzolari A.; Scaccabarozzi A.; Vistoli G.; Pedretti A. MetaClass, a Comprehensive Classification System for Predicting the Occurrence of Metabolic Reactions Based on the MetaQSAR Database. Molecules 2021, 26, 5857 10.3390/molecules26195857. 34641400 PMC8512547

[ref44] van Nifterik K. A.; van den Berg J.; van der Meide W. F.; Ameziane N.; Wedekind L. E.; Steenbergen R. D.; Leenstra S.; Lafleur M. V.; Slotman B. J.; Stalpers L. J.; Sminia P. Absence of the Mgmt Protein as Well as Methylation of the MGMT Promoter Predict the Sensitivity for Temozolomide. Br. J. Cancer 2010, 103, 29–35. 10.1038/sj.bjc.6605712. 20517307 PMC2905289

[ref45] Adhikari P.; Xie B.; Semeano A.; Bonifazi A.; Battiti F. O.; Newman A. H.; Yano H.; Shi L. Chirality of Novel Bitopic Agonists Determines Unique Pharmacology at the Dopamine D3 Receptor. Biomolecules 2021, 11, 11 10.3390/biom11040570. PMC8069330 33924613

[ref46] Pedretti A.; Mazzolari A.; Gervasoni S.; Fumagalli L.; Vistoli G. The Vega Suite of Programs: An Versatile Platform for Cheminformatics and Drug Design Projects. Bioinformatics 2021, 37, 1174–1175. 10.1093/bioinformatics/btaa774. 33289523

[ref47] Korb O.; Stützle T.; Exner T. E. Empirical Scoring Functions for Advanced Protein-Ligand Docking with Plants. J. Chem. Inf. Model. 2009, 49, 84–96. 10.1021/ci800298z. 19125657

[ref48] Vistoli G.; Mazzolari A.; Testa B.; Pedretti A. Binding Space Concept: A New Approach to Enhance the Reliability of Docking Scores and Its Application to Predicting Butyrylcholinesterase Hydrolytic Activity. J. Chem. Inf. Model. 2017, 57, 1691–1702. 10.1021/acs.jcim.7b00121. 28633528

[ref49] Santoni G.; Amantini C.; Nabissi M.; Maggi F.; Arcella A.; Marinelli O.; Eleuteri A. M.; Santoni M.; Morelli M. B. Knock-Down of Mucolipin 1 Channel Promotes Tumor Progression and Invasion in Human Glioblastoma Cell Lines. Front. Oncol. 2021, 11, 578928 10.3389/fonc.2021.578928. 33954107 PMC8092188

[ref50] Ricci-Vitiani L.; Pallini R.; Biffoni M.; Todaro M.; Invernici G.; Cenci T.; Maira G.; Parati E. A.; Stassi G.; Larocca L. M.; De Maria R. Tumour Vascularization Via Endothelial Differentiation of Glioblastoma Stem-Like Cells. Nature 2010, 468, 824–828. 10.1038/nature09557. 21102434

[ref51] Kleihues P.; Cavenee W. K. Pathology & Genetics. Tumours of the Nervous System. In World Health Organisation Classification of Tumours; IARC Press: Edinburgh, U.K., 2000, p 314.

[ref52] Visconti P.; Parodi F.; Parodi B.; Casarino L.; Romano P.; Buccarelli M.; Pallini R.; D’Alessandris Q. G.; Montori A.; Pilozzi E.; Ricci-Vitiani L. Short Tandem Repeat Profiling for the Authentication of Cancer Stem-Like Cells. Int. J. Cancer 2021, 148, 1489–1498. 10.1002/ijc.33370. 33128777 PMC7894552

[ref53] Agas D.; Amaroli A.; Lacava G.; Yanagawa T.; Sabbieti M. G. Loss of P62 Impairs Bone Turnover and Inhibits PTH-Induced Osteogenesis. J. Cell. Physiol. 2020, 235, 7516–7529. 10.1002/jcp.29654. 32100883

