# Supplementary material for: Highly Potent and Selective Dopamine D4 Receptor Antagonists Potentially Useful for the Treatment of Glioblastoma
Source: J Med Chem. 2022 Sep 13;65(18):12124–39. doi: 10.1021/acs.jmedchem.2c00840 (PMC9511495; doi:10.1021/acs.jmedchem.2c00840)
Supplement: Supplementary file 1 — jm2c00840_si_001.pdf [file jm2c00840_si_001.pdf]

SUPPORTING INFORMATION

Highly Potent and Selective Dopamine D_4 Receptor Antagonists Potentially Useful for the Treatment of Glioblastoma

Pegi Pavletić,† Ana Semeano,‡ Hideaki Yano,‡ Alessandro Bonifazi,# Gianfabio Giorgioni,† Alessandro Piergentili,*,† Wilma Quaglia,*,† Maria Giovanna Sabbieti,§ Dimitrios Agas,§ Giorgio Santoni,† Roberto Pallini,⊥,° Lucia Ricci-Vitiani,& Emanuela Sabato,^ Giulio Vistoli,^ Fabio Del Bello.†

†Scuola di Scienze del Farmaco e dei Prodotti della Salute, Università di Camerino, Via Madonna delle Carceri, 62032 Camerino, Italy

‡Department of Pharmaceutical Sciences, School of Pharmacy and Pharmaceutical Sciences, Bouvé College of Health Sciences, Center for Drug Discovery, Northeastern University, Boston, MA 02115, USA

#Medicinal Chemistry Section, Molecular Targets and Medications Discovery Branch, National Institute on Drug Abuse – Intramural Research Program, National Institutes of Health, 333 Cassell Drive, Baltimore, Maryland 21224, United States

§Scuola di Bioscienze e Medicina Veterinaria, Università di Camerino, Via Gentile III da Varano, 62032 Camerino, Italy

⊥Institute of Neurosurgery, Gemelli University Polyclinic Foundation, Scientific Hospitalization and Care Institute (IRCCS), Rome, 00168, Italy

°Institute of Neurosurgery, School of Medicine, Catholic University, Rome, 00168, Italy

&Department of Hematology, Oncology and Molecular Medicine, Istituto Superiore di Sanità, 00161 Rome, Italy

^Dipartimento di Scienze Farmaceutiche, Università degli Studi di Milano, Via Mangiagalli 25, 20133 Milano, Italy

*A.P.: e-mail: alessandro.piergentili@unicam.it; W.Q.: e-mail wilma.quaglia@unicam.it.

Table of Contents:

Table S1: Elemental analysis results for compounds **7-33**.

Table S2: Physico-chemical descriptors for compounds **5-33**.

Figure S1: HPLC chromatograms and experimental details for compound **24**.

Figure S2: HPLC chromatograms and experimental details for compound **29**.

Table S1. Elemental analysis results for compounds **7-33**.

**Compd**	**Formula**	**Calcd**				**Found**			
		**C%**	**H%**	**N%**	**S%**	**C%**	**H%**	**N%**	**S%**
**7**	$C_{21}H_{24}N_3.H_2C_2O_4$	67.46	6.65	10.26		67.21	6.78	10.11	
**8**	$C_{20}H_{24}N_4.H_2C_2O_4$	64.37	6.38	13.65		64.66	6.17	13.82	
**9**	$C_{20}H_{24}N_4.H_2C_2O_4$	64.37	6.38	13.65		64.12	6.29	13.43	
**10**	$C_{20}H_{23}N_3O.H_2C_2O_4$	64.22	6.12	10.21		63.99	6.36	10.34	
**11**	$C_{20}H_{23}N_3S.H_2C_2O_4$	61.81	5.89	9.83	7.50	62.11	7.30	6.10	7.27
**12**	$C_{22}H_{29}N_3.H_2C_2O_4$	67.74	7.34	9.88		67.44	7.19	9.99	
**13**	$C_{21}H_{25}N_3O.H_2C_2O_4$	62.99	7.93	10.02		63.25	7.90	10.19	
**14**	$C_{23}H_{29}N_3O.H_2C_2O_4$	66.21	6.89	9.27		65.27	6.54	9.36	
**15**	$C_{24}H_{31}N_3O.H_2C_2O_4$	66.79	7.11	8.99		66.41	7.02	9.09	
**16**	$C_{23}H_{29}N_3O.H_2C_2O_4$	66.21	6.89	9.27		65.88	6.83	9.08	
**17**	$C_{23}H_{29}N_3O.H_2C_2O_4$	66.21	6.89	9.27		66.10	6.81	9.33	
**18**	$C_{23}H_{29}N_3O.H_2C_2O_4$	66.21	6.89	9.27		66.34	6.96	9.34	
**19**	$C_{23}H_{29}N_3O_2.H_2C_2O_4$	63.95	6.66	8.95		64.11	6.47	9.12	
**20**	$C_{23}H_{29}N_3O_2.H_2C_2O_4$	63.95	6.66	8.95		63.74	6.80	8.92	
**21**	$C_{23}H_{29}N_3O_2.H_2C_2O_4$	63.95	6.66	8.95		63.90	6.41	8.76	
**22**	$C_{22}H_{26}ClN_3O.H_2C_2O_4$	60.82	5.96	7.48		60.74	5.83	7.60	
**23**	$C_{22}H_{26}ClN_3O.H_2C_2O_4$	60.82	5.96	7.48		60.52	6.01	7.61	
**24**	$C_{22}H_{26}ClN_3O.H_2C_2O_4$	60.82	5.96	7.48		61.02	6.06	7.50	
**25**	$C_{22}H_{26}N_4O_3.H_2C_2O_4$	59.50	5.83	11.56		59.59	5.70	11.80	
**26**	$C_{22}H_{26}N_4O_3.H_2C_2O_4$	59.50	5.83	11.56		59.38	5.83	11.34	
**27**	$C_{22}H_{26}N_4O_3.H_2C_2O_4$	59.50	5.83	11.56		59.29	5.98	11.40	
**28**	$C_{23}H_{26}N_4O.H_2C_2O_4$	64.64	7.00	14.96		64.78	7.01	15.20	
**29**	$C_{21}H_{26}N_4O.H_2C_2O_4$	62.71	6.08	12.06		62.99	6.31	12.31	
**30**	$C_{20}H_{25}N_5O.H_2C_2O_4$	59.85	6.16	15.86		59.98	6.00	16.03	
**31**	$C_{22}H_{25}Cl_2N_3O.H_2C_2O_4$	56.70	5.35	8.27		56.60	5.24	8.38	
**32**	$C_{22}H_{25}Cl_2N_3O.H_2C_2O_4$	56.70	5.35	8.27		56.51	5.31	8.12	
**33**	$C_{22}H_{25}Cl_2N_3O.H_2C_2O_4$	56.70	5.35	8.27		56.62	5.20	8.38	

Table S2. Physico-chemical descriptors for compounds **5-33**.

Compd	Rotors	HbAcc	HbDon	PSA	LogP	MW
**5**	7	2	0	24.85	4.38	328.49
**6**	5	2	0	28.95	3.17	349.47
**7**	5	1	0	11.37	4.11	319.44
**8**	5	2	0	22.55	3.24	320.43
**9**	5	2	1	34.65	4.44	320.43
**10**	5	3	0	30.51	4.83	321.42
**11**	5	2	0	42.00	4.37	337.48
**12**	5	1	0	11.74	4.91	335.49
**13**	4	2	0	27.66	2.77	335.44
**14**	6	2	0	27.49	3.61	363.50
**15**	7	2	0	28.56	3.84	377.52
**16**	5	2	0	27.46	3.45	363.50
**17**	5	2	0	27.68	3.58	363.50
**18**	5	2	0	28.21	3.60	363.50
**19**	6	3	0	38.97	3.34	379.50
**20**	6	3	0	40.59	3.25	379.50
**21**	6	3	0	38.97	3.28	379.50
**22**	5	2	0	28.21	3.77	383.91
**23**	5	2	0	28.01	3.76	383.91
**24**	5	2	0	27.64	3.71	383.91
**25**	5	4	0	66.88	3.67	394.47
**26**	5	4	0	68.32	3.71	394.47
**27**	5	4	0	69.24	3.58	394.47
**28**	5	2	0	47.04	2.47	374.48
**29**	5	3	0	37.35	2.21	350.46
**30**	5	4	0	47.96	1.58	351.45
**31**	5	2	0	27.64	4.32	418.36
**32**	5	2	0	28.57	4.28	418.36
**33**	5	2	0	28.02	4.33	418.36

A)

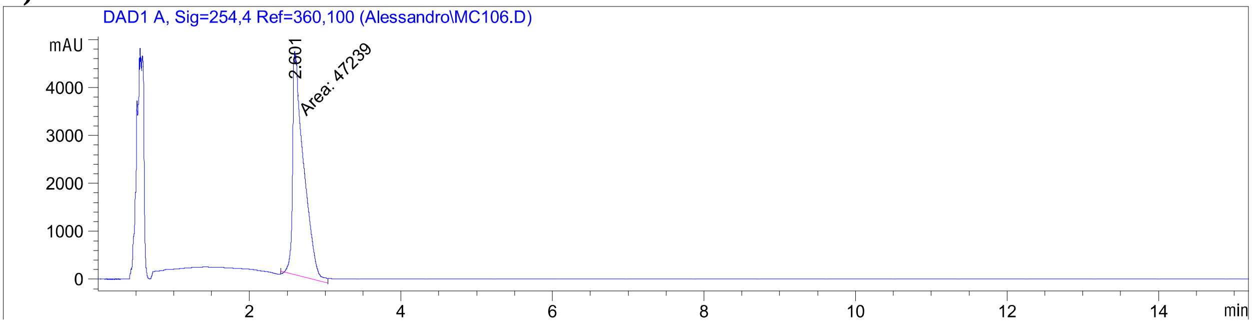

B)

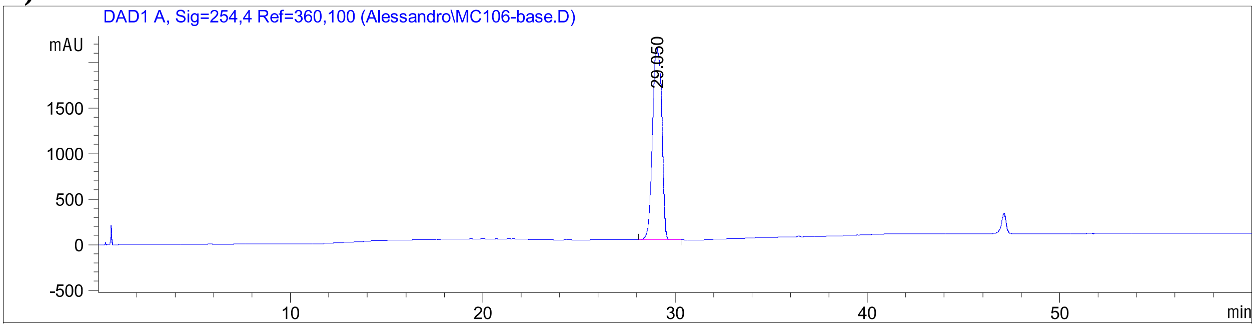

Figure S1. HPLC chromatograms of **24**. HPLC analysis was performed using an Agilent Technologies 1260 Infinity system coupled with DAD (Diode Array Detector). For each analytical HPLC run multiple DAD λ absorbance signals were measured in the range of 210-280 nm (representative chromatograms reported at λ254 nm). Separation of the analyte was achieved using a Phenomenex Gemini C18 4.6 x 50 mm. 3 μm column. **Method A)** mobile phase isocratic 30% ACN in water + 0.1% TFA; 15 min run; injection 20 μL (0.5 mg/mL); temperature 40 C; purity >95%; **Method B)** mobile phase gradient 10%-80% ACN in water + 0.1% DEA; 60 min run; injection 20 μL (0.5 mg/mL); temperature 40 C; purity >95%.

A)

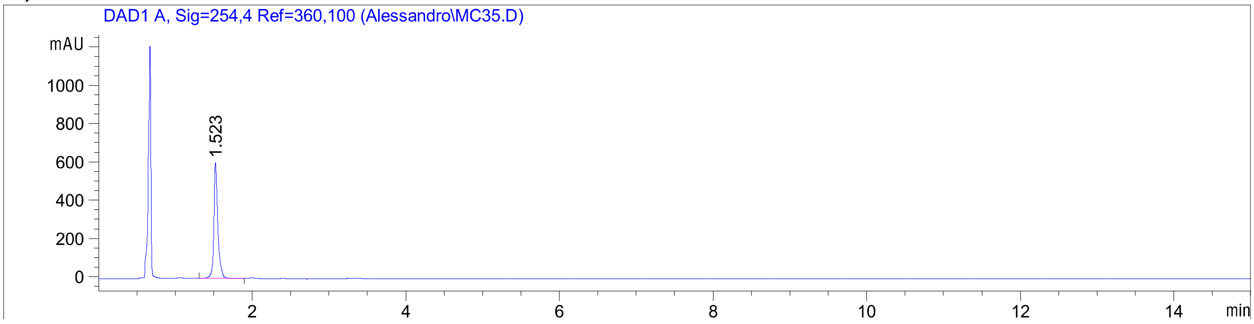

B)

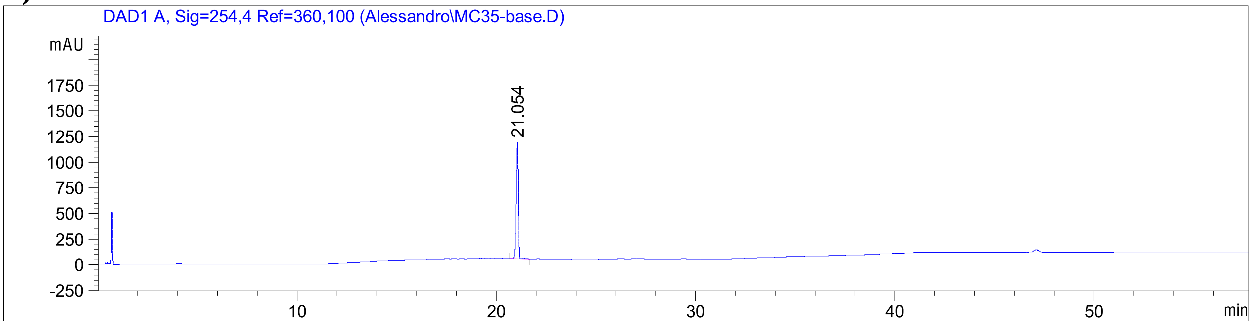

Figure S2. HPLC chromatograms of **29**. HPLC analysis was performed using an Agilent Technologies 1260 Infinity system coupled with DAD (Diode Array Detector). For each analytical HPLC run multiple DAD λ absorbance signals were measured in the range of 210-280 nm (representative chromatograms reported at λ254 nm). Separation of the analyte was achieved using a Phenomenex Gemini C18 4.6 x 50 mm. 3 µm column. **Method A)** mobile phase isocratic 15% ACN in water + 0.1% TFA; 15 min run; injection 20 µL (0.5 mg/mL); temperature 40 C; purity >95%; **Method B)** mobile phase gradient 10%-80% ACN in water + 0.1% DEA; 60 min run; injection 20 µL (0.5 mg/mL); temperature 40 C; purity >95%.
